# Synthesis of Nucleoside-like Molecules from a Pyrolysis Product of Cellulose and Their Computational Prediction as Potential SARS-CoV-2 RNA-Dependent RNA Polymerase Inhibitors

**DOI:** 10.3390/ijms23010518

**Published:** 2022-01-04

**Authors:** Andrea Defant, Federico Dosi, Nicole Innocenti, Ines Mancini

**Affiliations:** Laboratorio di Chimica Bioorganica, Dipartimento di Fisica, Università di Trento, Via Sommarive 14, 38123 Trento, Italy; fede.dosi@gmail.com (F.D.); nicole.innocenti@unitn.it (N.I.)

**Keywords:** anhydrosugar, nucleoside, Mitsunobu reaction, coronavirus, SARS-CoV2, molecular docking, molecular dynamics simulation, ADME prediction

## Abstract

(1*R*,5*S*)-1-Hydroxy-3,6-dioxa-bicyclo[3.2.1]octan-2-one, available by an efficient catalytic pyrolysis of cellulose, has been applied as a chiral building block in the synthesis of seven new nucleoside analogues, with structural modifications on the nucleobase moiety and on the carboxyl- derived unit. The inverted configuration by Mitsunobu reaction used in their synthesis was verified by 2D-NOESY correlations, supported by the optimized structure employing the DFT methods. An in silico screening of these compounds as inhibitors of SARS-CoV-2 RNA-dependent RNA polymerase has been carried out in comparison with both remdesivir, a mono-phosphoramidate prodrug recently approved for COVID-19 treatment, and its ribonucleoside metabolite GS-441524. Drug-likeness prediction and data by docking calculation indicated compound **6** [=(3*S*,5*S*)-methyl 5-(hydroxymethyl)-3-(6-(4-methylpiperazin-1-yl)-9H-purin-9-yl)tetrahydrofuran-3-carboxylate] as the best candidate. Furthermore, molecular dynamics simulation showed a stable interaction of structure **6** in RNA-dependent RNA polymerase (RdRp) complex and a lower average atomic fluctuation than GS-441524, suggesting a well accommodation in the RdRp binding pocket.

## 1. Introduction

Cellulose is the most biosynthesized organic substance on earth and is an abundant component of plants. According to the biorefinery concept, it represents a key biomass to produce fuels and chemicals. In line with the sustainability of bioresources, the conversion of the carbohydrate content in lignocellulosic waste represents an interesting valorization. Biomass-derived compounds are often rich in functionalization and stereocenters, so that to be crucial chiral pools for the synthesis of non-racemic molecules. 

**Figure 1:**
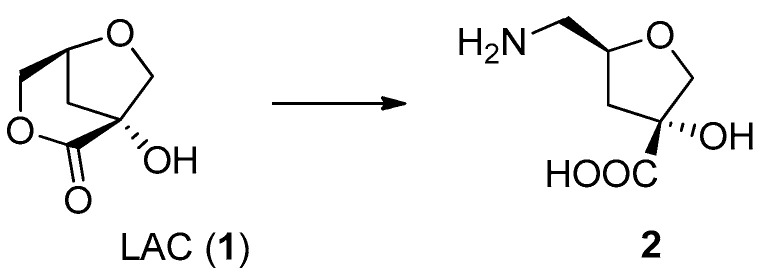
Molecular structures of LAC from cellulose pyrolysis and of the amino acid (3*R*,5*S*)-5-(amino-ethyl)-3-hydroxytetrahydrofurane-3-carboxylic acid (**2**).

The bio-oil produced by catalytic pyrolysis of cellulose contains anhydromonosaccharides and among them the hydroxylactone LAC (**1**) (=(1*R*,5*S*)-1-hydroxy-3,6-dioxa-bicyclo[3.2.1]octan-2-one) [1] in Figure 1 turned out to be a compound with added value as chiral building block to be used in asymmetric synthesis. In particular, an improved production on the gram scale by pyrolysis in the presence of cheap and eco-friendly catalyst [2] has favored the use of LAC to obtain enantiomerically pure bioactive molecules. This was used to produce the new branched δ-sugar amino acid **2** (Figure 1) acting as a conformationally restricted isostere of the glycine–alanine dipeptide with potential applications in peptidomimetics [3]. Furthermore, the structural similarity of this amino acid with L(+)-muscarine inspired us the production of a series of new compounds showing affinity for human cloned muscarinic receptors [4].

**Figure 2:**
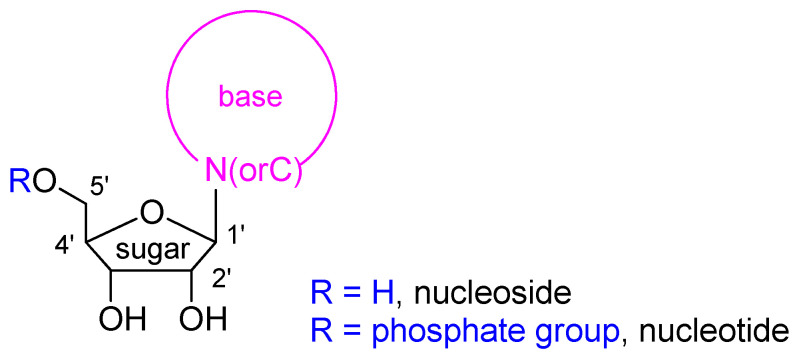
General molecular structure of N- or C- nucleosides/nucleotides.

In the present work LAC is used to obtain nucleoside-like molecules with potential biological activities. Nucleosides consist of a nucleobase, typically a purine or pyrimidine, and a five-carbon sugar, displaying a remarkable chemical diversity in nucleoside-based secondary metabolites. Both natural and synthetic nucleosides and nucleotides (Figure 2) exhibit peculiar biological properties. Nucleosides or their analogues are used in the treatment of cancer and viral infections. Regarding their antiviral application, over 25 nucleoside and nucleotide analogues were approved as therapeutic agents [5]. The majority of them target enzymes involved in virus replication, including RNA viral polymerases which have proven to be valid targets for the development of antiviral agents, because all RNA viruses encode an RNA-dependent RNA polymerase (RdRp). Nucleotide and nucleoside inhibitors are usually administered as prodrugs, which are metabolized to their active triphosphate once inside the cell [6].

In the last few years, the disease causing the severe acute respiratory syndrome coronavirus-2 (SARS-CoV-2), named COVID-19, has spread globally, causing a public health emergency. Vaccines aim to prevent infection and a series of vaccines have been made available to fight this pandemic relatively quickly. However, there is also the pressing need to develop additional effective therapy. The use of antibodies is proving to be a valid treatment in some cases and antiviral drugs can offer an effective remedy to treat the worst symptoms, especially for immunosuppressed people. Potential repurposed antiviral drugs are currently under evaluation, including agents active against HIV, Ebola and Zika infections [7].

**Figure 3:**
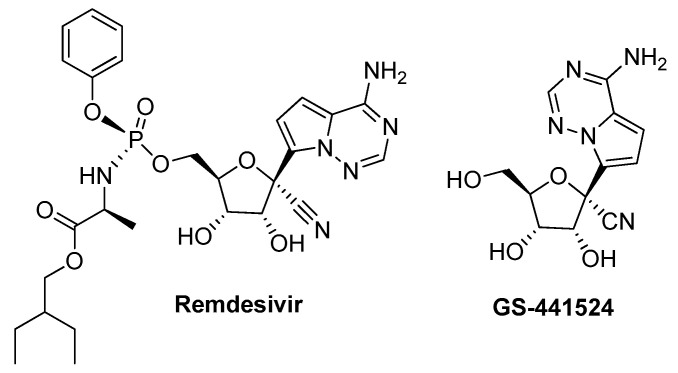
Molecular structure of remdesivir and GS-441524.

Remdesivir (GS-5734, Figure 3) is active against a series of viruses, by inhibiting RdRp to stop viral replication [8]. Recently, it has been approved to treat COVID-19 by the United States Food and Drug Administration (FDA). Acting as a monophosphate prodrug, remdesivir is the precursor of the 10-cyano adenosine nucleoside GS-441524 (Figure 3), because after administering it is subjected to an in vivo bioactivation, producing GS-441524 as the predominant metabolite circulating in the bloodstream [9]. Displaying a higher efficacy than remdesivir and several advantages [9], it has also been authorized for COVID-19 treatment. 

We report here on the synthesis of a series of new nucleoside-like molecules starting from the chiral building block LAC available from an efficient ecofriendly catalytic pyrolysis of cellulose. The drug-likeness properties of these products have been evaluated obtaining a favorable pharmacokinetic profile and the related phosphate compounds have been studied in silico as inhibitors of SARS-CoV-2 RNA-dependent RNA polymerase providing promising results when compared with remdesivir and its metabolite.

## 2. Results and Discussion

### 2.1. Synthesis of Nucleosides **5**–**11**

**Scheme 1:**
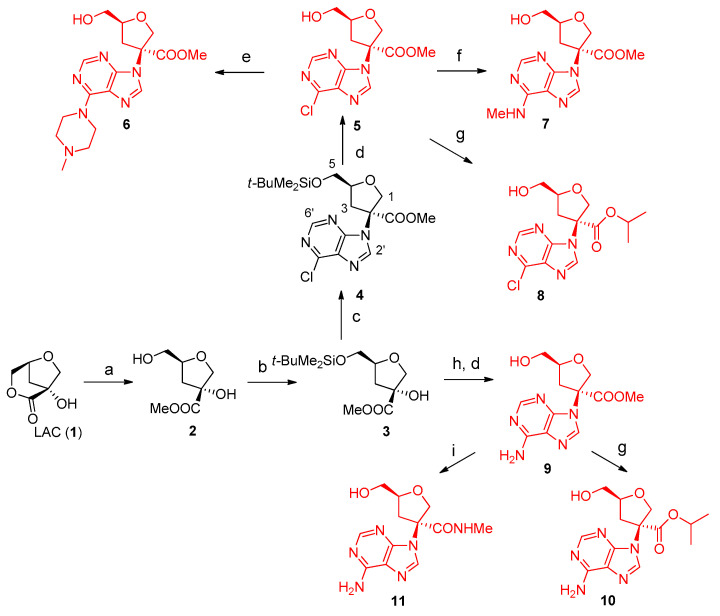
Synthesis of nucleosides **5**–**11** from LAC. Reagents and conditions: (**a**) MeOH/Et_3_N (2.5 eq), r.t., 12 h, 95%; (**b**) *t*-BuMe_2_SiCl, imidazole, CH_2_Cl_2_, r.t., 2 h, 78%; (**c**) DEAD, Ph_3_P, 6-chloropurine, THF, reflux 24 h, 48%; (**d**) TBAF, THF, r.t., 2 h, 87%; (**e**) *N*-methylpiperazine, MeOH, *M*_W_, 70 °C, 1.5 h; 75%; (**f**) CH_3_NH_2_ soln., MeOH, *M*_W_, 70 °C, 20 min. 92%; (**g**) *i*-PrOH, TBD, *M*_W_, 50 °C, 30 min, 53%; (**h**) DEAD, Ph_3_P, adenine, THF, reflux 10 h, 60%; (**i**) CH_3_NH_2_ soln., MeOH, 0 °C, r.t. overnight, 60%. Arbitrary numbering is for convenience.

Seven nucleoside analogues were synthesized from the same common precursor **3**, available by protecting the primary alcohol as tertbutyldimethylsilyloxy derivative of the methyl ester **2** accessible from the lactone LAC (**1**, Scheme 1). Structural modifications are introduced on the nucleobase moiety (Figure 2) (as chloropurine in **5**, **8**, adenine in **9**–**11**, methylpiperazinyl purine in **6**, methylaminopurine in **7**), and on the carboxyl-derived unit (methyl ester in **5**–**7** and **9**, *i*-propyl ester in **8** and **10** and *N*-methylamide in **11**).

**Figure 4:**
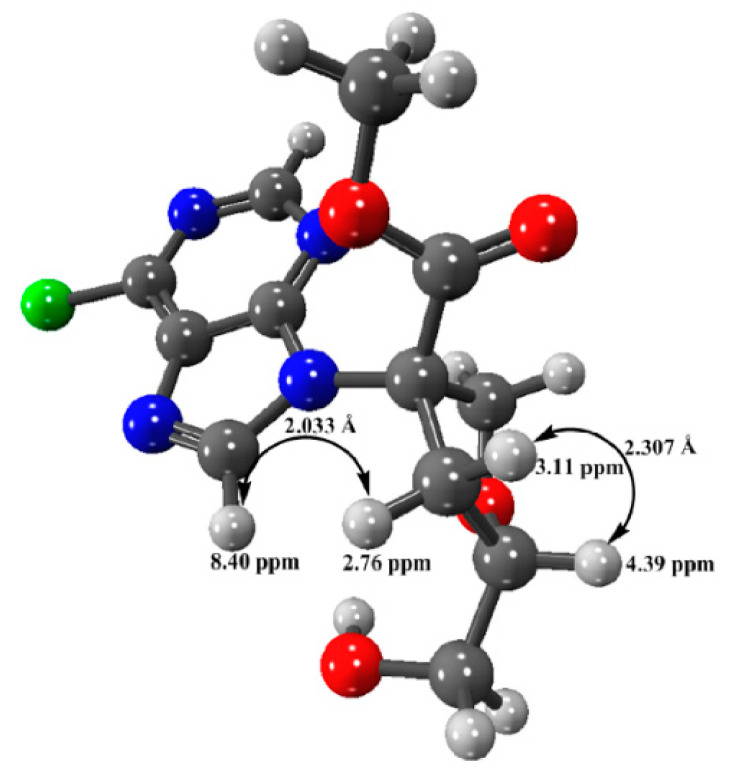
Experimental NOE correlations observed for compound **5**, supported by the energy-minimized structure shown here, obtained by DFT calculation in chloroform.

In detail, compound **4** was obtained by a Mitsunobu reaction of **3** with 6-chloropurine in THF using diethyl azodicarboxylate (DEAD) and triphenylphosphine [10]. By treating **4** with tetrabutylamoniun fluoride, the compound **5** was produced. Based on Mitsunobu mechanism, a good leaving group is generated by reaction of the alcohol with triphenylphospine followed by a bimolecular nucleophilic substitution providing inversion of stereochemistry. 2D-NOESY experiment acquired for compound **5** in CDCl_3_ confirmed the derived (*S*) configuration at C-2, starting from the (*R*)-assigned in LAC. A correlation was observed between proton at 4.39 ppm assigned at C-4 position (of known (*S*) configuration based on LAC as starting chiral building block) with H-3 at 3.11 ppm, and for the other proton in position 3 at 2.76 ppm with the singlet at 8.40 ppm assigned to H-2′ in purine unit. Evidence from these experiments found support in the energy-minimized structure of **5** by density functional theory (DFT) calculation in chloroform, where the distances (2.307 Å and 2.033 Å, respectively) are compatible with the observed NOE (Figure 4). The *N*-methylpiperazinyl moiety in analogue **6** and the *N*-methyl unit in derivative **7** were efficiently provided in good yields by treating the chloropurinyl compound **5** with the corresponding amines under microwave irradiation (*M*_W_) in methanol. Similar technique was adopted to obtain analogue **8** by triazabicyclodecene (TBD)-catalyzed transesterification of the methyl ester **5** with *i*-propanol. As derivatives from **5**, the absolute configurations of the analogues **6**–**8** were established. The precursor **3** was also used to produce compound **9** by a first Mitsunobu reaction with adenine, followed by alcohol deprotection, similarly to the method previously adopted to obtain **5**. The TBD-catalyzed substitution with *i*-propanol converted the methyl ester **9** to the analogue **10**. Product **11** could be also obtained by reaction of **9** with a solution of methylamine. The inversion of configuration by the Mitsunobu reaction ascertained for **5** can also be assumed for **9**, therefore **9** itself and its derivatives **10** and **11** have known absolute configurations.

### 2.2. Pharmacokinetics Studies

**Figure 5:**
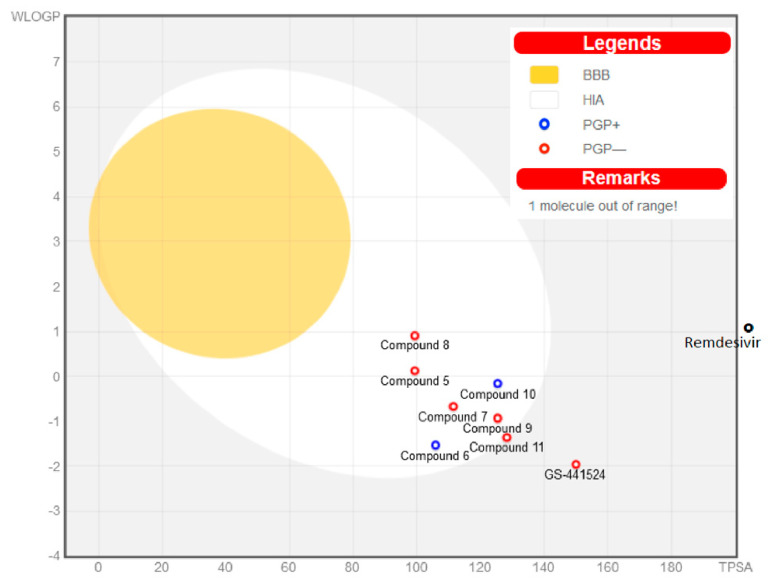
WLOGP-versus-TPSA boiled egg visualization compounds **5**–**11** in comparison with remdesivir and GS-441524.

In this virtual screening, the synthetic compounds **5**–**11** were evaluated for their pharmacokinetic parameters, drug-likeness and ADME analysis, in comparison with remdesivir and its metabolite GS-441524. Besides respecting Lipinski’s rule, the calculated pharmacokinetic parameters for all synthetic molecules (Table S1) are very promising, with a bioavailability score better than remdesivir (0.55 versus 0.17, Table S1). It is also pointed out in the bioavailability radar view (Figure S8), obtained by Swiss ADME server which takes into account six physicochemical properties: lipophilicity, size, polarity, solubility, flexibility and saturation. A correlation of the molecular structures with these parameters (Table S1) highlights that (i) the lipophilicity is affected by the alkyl ester: the *i*-propyl unit increases the values as evident by comparison the corresponding analogues **5**/**8** and **9**/**10**, as well as the presence of N-methylamide reduces the lipophilicity observed for the corresponding esters **9** and **10**; the replacement of chlorine with an amino group is able to decrease the values in the order **5** > **7** ≈ **6** > **9** and **8** > **10**, respectively; (ii) the polarity, expressed as topological polar surface area (TPSA) is in the range 99–128 Å^2^, to be compared with a value of 150 Å^2^ for GS-441524 and of 213 Å^2^ for remdesivir, and indicating a very favorable parameter for the compounds **5**–**11**; (iii) the water solubility descriptors in the order **11** > **5**–**7**, **9**, **10**, GS-441524 > **8** >> remdesivir, pointing out the potential of LAC-derived molecules as orally administered drugs. In addition, a high gastrointestinal (GI) absorption [11] for **5**–**10** (and low for **11**, remdesivir and GS-441524) is predicted, as displayed in the white region of boiled-egg view (Figure 5). Drug-likeness model score computed by MolSoft server considers a combined effect of physicochemical properties, pharmacokinetics and pharmacodynamics of a compound, given as a numerical value by comparison with 5000 market drugs (assigned positive value) and 10,000 molecules with non-drug properties (assigned negative values). In the evaluation of compounds **5**–**11**, remdesivir and its metabolite, a wide variability was found, with the highest drug-likeness score (0.95) for compound **6** (Figure S9).

### 2.3. Docking Calculation and Molecular Dynamics

**Figure 6:**
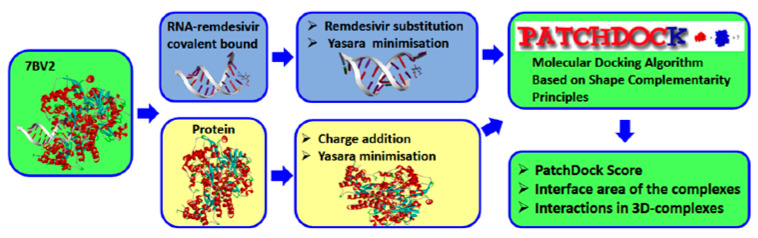
Workflow of the computational analysis.

RdRp was selected as a drug target because this protein is essential for viral replication and transcription of SARS-CoV-2. Our computational approach took into account the potential inhibition by template-primer RNA covalently linked to remdesivir or the ligands **5**–**11** here investigated (Figure 6). The process started from the nsp12-nsp7-nsp8 complex bound to the template-primer RNA and phosphate form of remdesivir structure deduced by cryogenic electron microscopy (cryo-EM) analysis at 2.5 Å of resolution (PDB ID: 7BV2). Protein and RNA-remdesivir were split, remdesivir was replaced by each monophosphate form of **5**–**11** and the obtained structure minimized by Yasara software. The free protein was optimized and docked with RNA by PatchDock server. The approach we have adopted in docking calculation is noteworthy, especially if related with similar systems reported in the literature. Recently, an in silico evaluation on the interaction of the full structure of remdesivir in the catalytic site of RdRp in the free form has been reported [12]. However, the mechanism of action was known for remdesivir acting as a prodrug to produce GS-441524 monophosphate, which is subsequently converted into the triphosphorylated form, able to be incorporated by the SARS-CoV-2 RdRp complex [8]. Furthermore, the RdRp considered (PDB ID 7BTF) contains no primer-RNA binding in the catalytic pocket, providing unreliable results. Conversely, we have applied a more rigorous method starting from a cryo-EM deduced structure [13] which contains template-primer RNA and monophosphate form of GS-441524 (PDB ID 7BV2) and available with a higher resolution in comparison with 7BTF. This establishes the active pocket for the binding of ligands. The presence of two interacting macromolecules resulted not easy treating them with a simple docking protocol. The approach included a first step where each single structure of template-primer RNA after replacement of GS-441524 with every molecule **5**–**11** was minimized by molecular mechanics, followed by application of a molecular docking algorithm based on shape complementary principles.

This computational approach has provided the docking score and interface area values reported in Table 1. Higher values in both the geometric descriptor of shape complementarity [14] and approximate interface area reflect a better interaction in the complex. All molecules **5**–**11** showed higher values of both geometric shape score and interface area than GS-441524 (Table 1).

**Figure 7:**
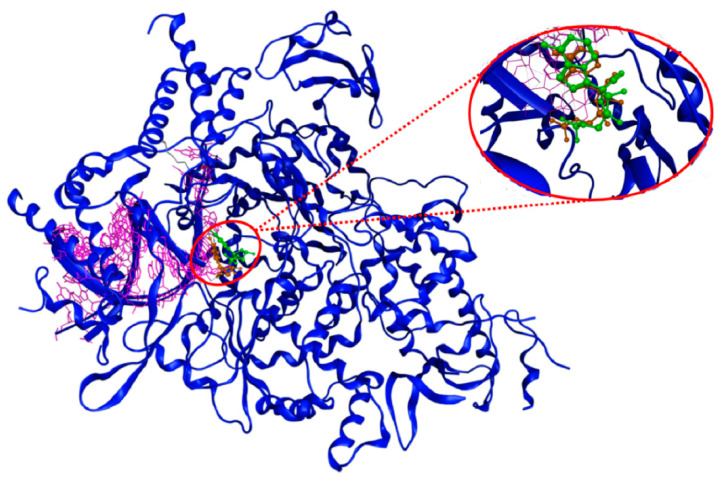
Overlapping of X-ray derived structure of RdRp (7BV2) and PatchDock results of the complex with GS-441524. Ribbon structure of Protein and RNA in blue, atoms of RNA in magenta, GS-441524 covalently linked by X-ray analysis in green, GS-441524 covalently linked by docking calculation in orange.

As displayed in Figure 7, the results obtained by rigid docking calculation were in agreement with experimental data, validating the unconventional approach herein adopted for the particularly complex investigated system.

**Figure 8:**
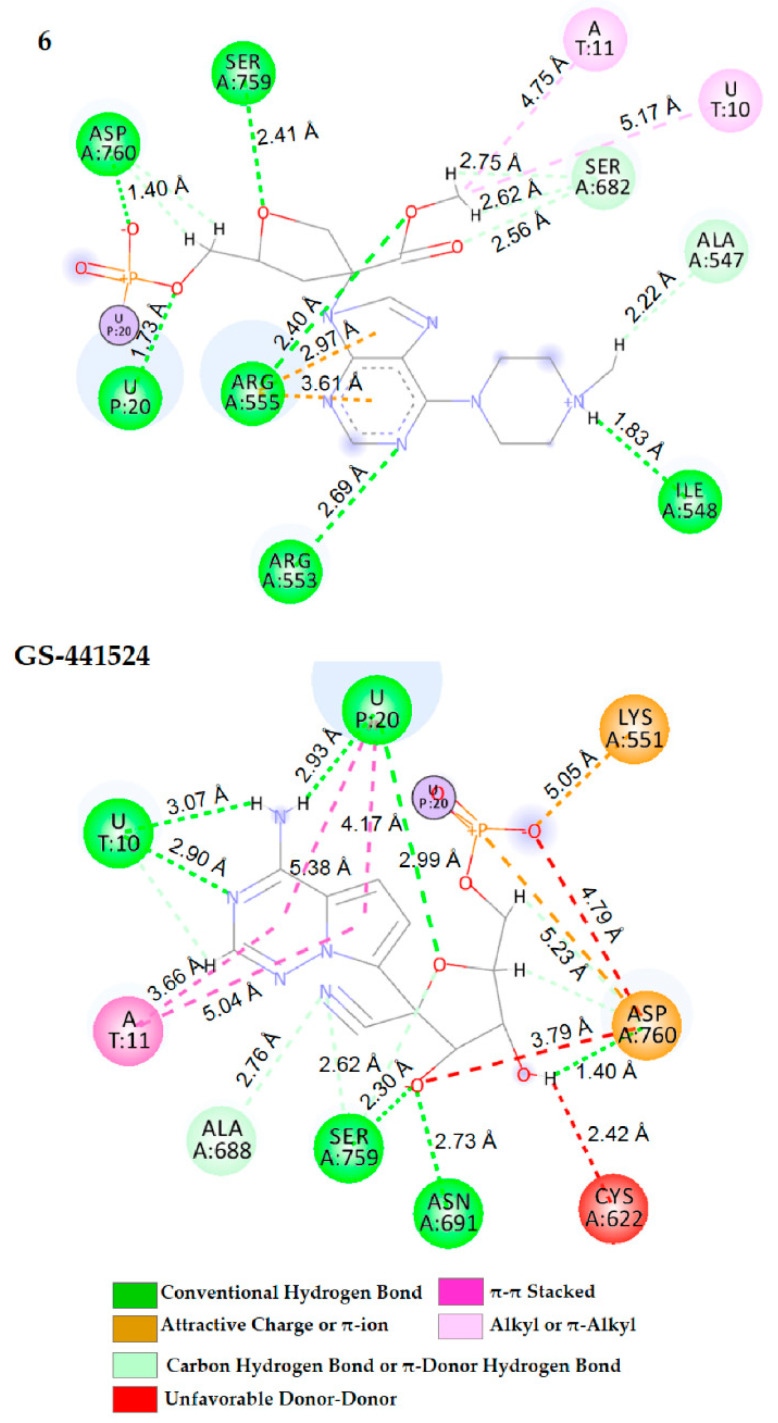
Two-dimensional representations for the interactions of both compound **6** and GS-441524 linked as monophosphate to RNA in the receptor pocket of free RdRp (7BV2) with indication of interaction distance, as deduced by docking calculations.

Table 2 summarizes the specific interactions obtained for each ligand compound in the corresponding complex. The type and number of interactions involving compound **6** are better than the other LAC-derivative molecules, and even better than GS-441524, with the latter also displaying unfavorable interactions. These results are illustrated in Figure 8 for compound **6** compared with GS-441524, both linked as monophosphate, and in Figure S10 for the compounds **5** and **7**–**11**.

This type of study explicitly states which of the amino acids are involved and to compare them with the standard drugs [15], in this case remdesivir metabolite. The results by docking calculation for compound **6** linked to primer RNA interacting in the enzyme pocket show strong H-bonds with Ile 548 (Ile-C=O and MeNH^+^ in *N*-methyl piperazinyl unit with a distance of 1.83 Å), Arg 553 (guanidine *N*H in Arg with purine N3 with a distance of 2.69 Å), Arg 555 (guanidine NH in Arg with OMe in ester unit, with a distance of 2.40 Å), Ser 759 (Ser-OH with O-tetrahydrofurane ring, with a distance of 2.41 Å) and Asp 760 (Asp-COOH Asp-*N*H both with O-phosphate with a distance of 1.18 Å and of 1.96 Å, respectively). Regarding the H-bonds involved for GS-441524 linked to primer RNA interacting in the pocket, Ser 759 (Ser-OH with 2′OH with a distance of 1.24 Å) is present similarly to compound **6**. Asp760 (Asp-COOH with 3′O with a distance of 1.40 Å) is another common amino acid, but also involved in two repulsive unfavorable interactions with O-phosphate and 3-OH). In addition, Asn 691 (Asn-CONH_2_ with 2′O, with a distance of 1.73 Å) and an unfavorable interaction with thiol unit in Cys 622. It is to note that the specific interactions involving the *N*-methyl piperazinyl unit with Ile 548 and the COOMe group for compound **6** are decisive for a better potential RdRp inhibition than for GS-441524.

In light of score data on geometric shape complementarity and interface area (Table 1) and both the number of interactions and the corresponding distance (Table 2), **6** resulted as the most interesting molecule and therefore was selected for molecular dynamics (MD) simulation. In order to evaluate the stability in the time of RdRp free and in each complex with a sequence of RNA bonded to compound **6** or GS-441524, a 35 ns simulation has been performed on each structure. The potential energy of free enzyme and the complex with remdesivir metabolite resulted stable for all the simulation time, whereas for complex containing compound **6** showed to be stable after around 7 ns of simulation (Figure S11). 

Another parameter establishing the quality of structure is Z-score [Z = (x − µ)/σ], where x represents the energy of current structure, µ and σ are the average value and the standard deviation of energy in the “gold standard” protein population. For all structures, Z resulted about −1.35, meaning that each of them was satisfactory (Figure S11). The radius of gyration (Rg), which is the mean-square mass-weighted root range of a set of atoms that shared the mass center [16] has been calculated. The Rg values for free RdRp and for both complexes showed similar values during all simulation time, very close to the one for the original X-ray protein, indicating a similar conformation flexibility (Figure S12).

For evaluating the thermodynamic stability of the system, Root Mean Square Deviation (RMSD) was calculated, both for Cα and backbone as well as for all the heavy atoms. Similar values of the protein in the starting and in complexed structures were obtained, in the range of 1.2–1.9 Å (Figure S12). In general, small values represent a minor flexibility in the structure [17,18].

**Figure 9:**
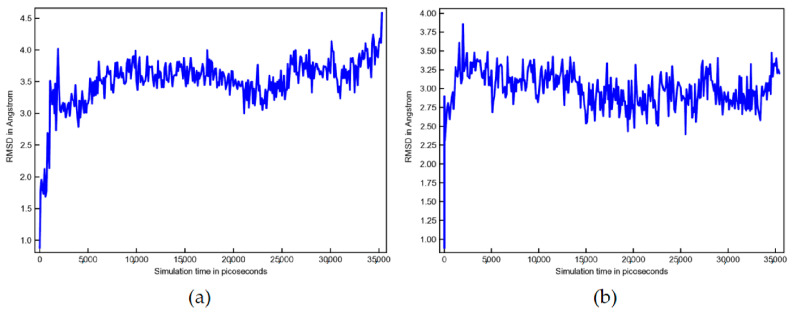
Root Mean Square Deviation (RMSD) graphs obtained by molecular dynamics simulation showing the variation as a function of time of both ligand movement for: (**a**) compound **6** and (**b**) GS-441524.

**Figure 10:**
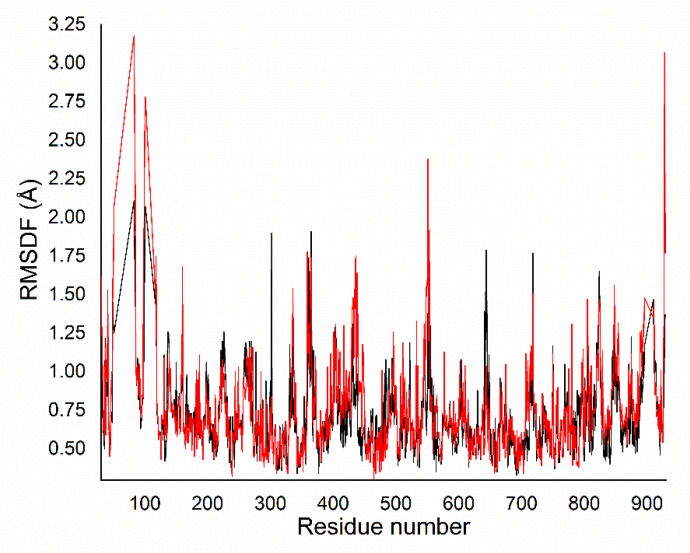
Root Mean Square Fluctuation (RMSF) for compound **6** (black line) and GS-441524 (red line).

Ligand movement RMSD after superposing on the receptor gives information on the movement of the ligand in its binding pocket. Average values 3.50 Å and 3.25 Å resulted for **6** and GS-441524, respectively (Figure 9). Regarding the fluctuation of amino acidic residues in the structure, Root Mean Square Fluctuation (RMSF) showed the involvement of the same amino acid residues, with very similar range of motion for both complexes under investigation (Figure 10).

**Figure 11:**
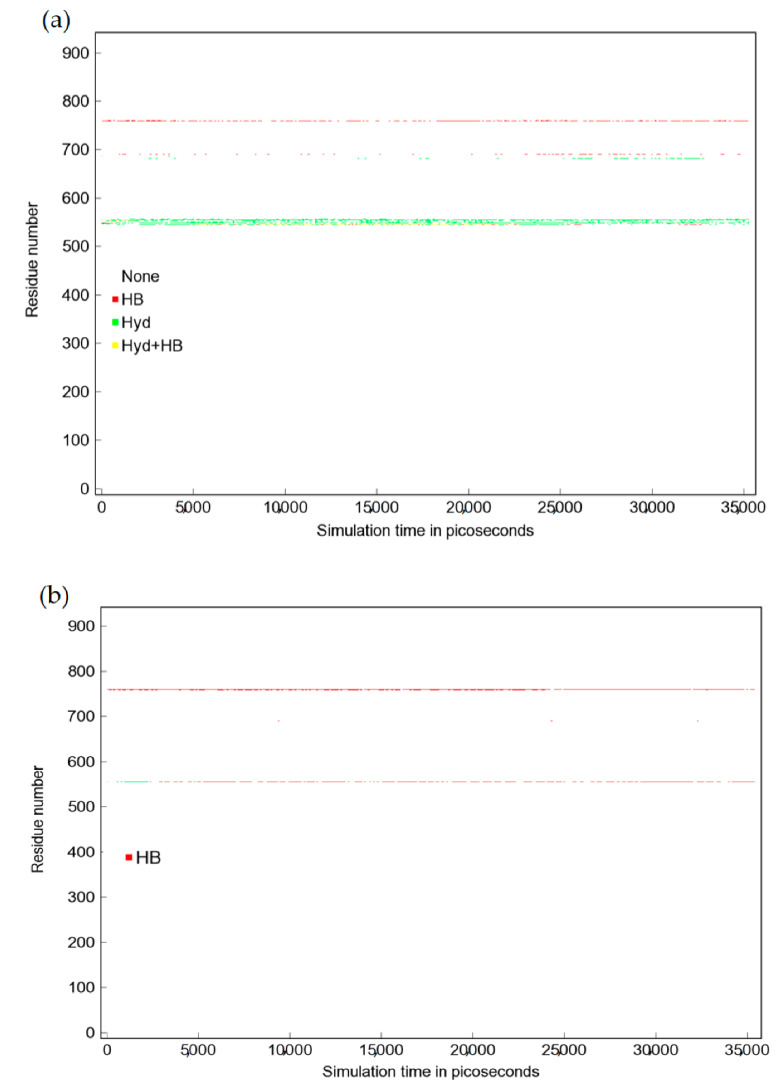
Per-residue contacts including H-bonding (in red) and hydrophobic (in green) interactions in function of the time involved for: (**a**) compound **6** and (**b**) GS-441524.

Figure 11 shows H-bonding and hydrophobic interactions during the simulation. In detail, for compound **6** strong H-bonds with Asp 760 and Ser 759 remain stable during all simulation, as depicted by an almost continuous sequence of the red line. In addition, H-bonds are established with Ile 548, Arg 553 and Arg 555 which are more labile during the time. This evidence is in line with the distance evaluated by docking calculation. A further unstable H-bond is sometime formed with Asn 691, as deduced in Yasara MD analysis by per-residue contacts with ligand table. Stable hydrophobic interactions are also present involving Ala 547, A11 and U10, whereas the hydrophobic interaction with Ser 682 becomes unstable. For the reference compound GS-441524, Ser 759, U10 and U20 are involved in stable H-bonds during the time, whereas Asn 691 is strongly unstable and is destroyed. On the contrary, Arg 555 forms a stable enough H-bond. Regarding GS-441524, H-bonds with Ser 759 and Asp 760 are involved, stable during all simulation, and a new fluctuating H-bond with Arg 555 is formed, not present from docking calculation. 

## 3. Materials and Methods

### 3.1. Chemistry

#### 3.1.1. General

All reagents were purchased from Sigma Aldrich (WVR, Milan, Italy) and used without further purification. Preparation of amino acid **2** from LAC was carried out according to the reported procedure [3]. Microwave assisted reactions were carried out using a Discover CEM microwave reactor. The reactions were not optimized and the yields were calculated for the products after chromatographic purification, based on the reacted starting material. Thin layer chromatography (TLC): Merck silica gel F254 or reversed phase Merck RP-18 F254 (WVR, Milan, Italy), with visualization using UV light. Flash chromatography (FC): Merck Si 15–25 µm (WVR, Milan, Italy). Preparative thin layer chromatography (PLC): 20 × 20 cm Merck Kieselgel 60 F254 0.5-mm plates (WVR, Milan, Italy). Polarimetric data were obtained using a JASCO-dip-181 apparatus, reporting [α]_D_ in dm^−1^·deg·mL·g^−1^. Infrared spectra were recorded by using a FT-IR Tensor 27 Bruker spectrometer (Attenuated Transmitter Reflection, ATR configuration) at 1 cm^−1^ resolution in the absorption region 4000–600 cm^–1^. A thin solid layer is obtained by evaporation of methanol solution of the sample. The instrument was purged with a constant dry air flux and clean ATR crystal as background was used. Spectra processing was made using Opus software package. NMR spectra were recorded on a Bruker-Avance 400 spectrometer using a 5-mm BBI probe ^1^H at 400 MHz and ^13^C at 100 MHz with values relative to TMS, in CDCl_3_ (δ_H_ 7.25 and δ_C_ 77.00 ppm) or acetone-d_6_ (δ_H_ 2.05 and δ_C_ 29 ppm) with δ values in ppm and *J* values in Hz; assignments were supported by polarization transfer (DEPT), heteronuclear single quantum correlation (HSQC) and heteronuclear multiple bond correlation (HMBC) experiments; nuclear Overhauser enhancement (nOe) data from bidimensional NOESY experiments. Electron impact (EI)–MS and high-resolution HR-EI-MS spectra (*m*/*z*; rel.%) were recorded with a Kratos MS80 mass spectrometer equipped with home-built computerized acquisition software. Electrospray ionization (ESI)-MS mass spectra were recorded using a Bruker Esquire-LC spectrometer by direct infusion of a methanol solution (source temperature 300 °C, drying gas nitrogen, 4 L·min^−1^, scan range *m*/*z* 100–1000). High-resolution ESI-MS measurements were obtained by direct infusion using an Orbitrap Fusion Tribrid mass spectrometer.

#### 3.1.2. Synthesis and Structural Characterization of Compounds **3**–**11**

1. *(3R,5S)-Methyl 3-hydroxy-5-(hydroxymethyl)tetrahydrofuran-3-carboxylate (**2**).* The compound was obtained following the procedure reported and structural assignment is in agreement with known data [3].
2. *(3R,5S)-Methyl 5-(((tert-butyldimethylsilyl)oxy)methyl)-3-hydroxytetrahydrofuran-3-carboxylate (**3**).* A solution of compound **2** (0.998 g, 5.67 mmol) in dichloromethane (20 mL) was stirred with *tert-*butylchlorodimethylsilane (1.20 g, 7.94 mmol) and imidazole (0.965 g, 14.18 mmol) at room temperature for 2 h, monitoring the total conversion by TLC. The reaction mixture was concentrated in vacuo and the residue subjected to FC on silica gel, using hexane/ethyl acetate gradient elution as eluent, obtaining pure **3** as a colorless oil. TLC (hexane/EtOAc = 40:60 *v*/*v*): Rf = 0.88. Yield: 78%. FT-IR (cm^−1^): 2952 (w), 1727 (s), 1463 (w), 1238 (s), 1099 (s), 837 (vs), 779 (s). ^1^H-NMR (400 MHz, CDCl_3_) δ 4.25 (m, 1H, H4), 4.13 (d, *J* = 9.5 Hz, 1H, H_a_1) 3.79 (d, *J* = 9.5 Hz, 1H, H_b_1), 3.78 (s, 3H, OMe), 3.73 (dd, *J* = 11.0, 3.9 Hz, 1H, H_a_ 5), 3.67 (dd, *J* = 11.0, 4.3 Hz, 1H, H_b_ 5), 2.30 (dd, *J* = 12.5, 9.4 Hz, 1H, H_a_ 3), 2.02 (dd, *J* = 12.5, 5.5 Hz, 1H, H_b_ 3), 0.85 (s, 9H, *t*Bu), 0.03 (s, 6H, SiMe_2_).^13^C-NMR (100 MHz, CDCl_3_) δ 181.3 (COO), 80.9, 79.7, 77.5 (C1), 67.9 (C5), 57.9 (OMe), 37.5 (C3), 28.3 (*t*Bu), 1.5 (SiMe_2_). EI-MS: *m*/*z* 290 (M^+^, 1), 233 (24), 159 (8), 73 (100).
3. *(3S,5S)-Methyl 5-(((tert-butyldimethylsilyl)oxy)methyl)-3-(6-chloro-9H-purin-9-yl)tetrahydrofuran-3-carboxylate (**4**).* Diethyl azodicarboxylate (DEAD, 40% in toluene, 1.442 g, 8.27 mmol) was slowly added to triphenylphosphine (2.168 g, 8.27 mmol) in THF (8 mL) under N_2_ atmosphere at 0 °C. After stirring for 30 min, 6-chloropurine (0.478 g, 3.10 mmol) and compound **3** (0.600 g, 2.068 mmol) in THF (8 mL) were added and the reaction mixture refluxed for 24 h. The solvent was evaporated and the crude residue was purified by column chromatography using hexane/EtOAc gradient elution to recover 230 mg of **3** and pure **4** (252 mg) as a light yellow oil TLC (hexane/EtOAc = 70:30 *v*/*v*): Rf = 0.20. Yield 48%. [α]_D_^20^= +20.0 (25.1 mg/mL, acetone). FT-IR (cm^−1^): 2954 (m), 2930 (m), 2858 (w), 1747 (s), 1589 (s), 1562 (s), 1486 (m), 1472 (m), 1437 (m), 1400 (m), 1343 (m), 1297 (w), 1257 (s), 1220 (s), 1149 (s), 1092 (m), 1006 (m), 936 (m), 837 (s), 779 (m). ^1^H NMR (400 MHz, CDCl_3_) δ 8.69 (s, 1H, H6′), 8.27 (s, 1H, H2′), 4.73 (d, *J* = 12 Hz, 1H, H_a_1) 4.47 (d, *J* = 12 Hz, 1H, H_b_1), 4.37 (m, 1H, H4), 3.71 (s, 3H, OMe), 3.69 (m,1H, H_a_5), 3.60 (dd, *J =* 11.2, 3.9 Hz, 1H, H_b_5), 3.16 (dd, *J* = 13.6, 7.9 Hz, 1H, H_a_3), 2.69 (dd, *J* = 13.6, 7.3 Hz, 1H, H_b_3), 0.74 (s, 9H, *t*Bu), −0.07 (s, 6H, SiMe_2_). ^13^C-NMR (100 MHz, CDCl_3_) δ 169.5 (COO), 152.0 (C6′), 151.6, 143.5 (C2′), 131.9, 80.2 (C4), 74.7(C1), 70.3, 63.7 (C5), 53.8 (OMe), 37.5 (C3), 25.1 (*t*Bu), −6.1 (SiMe_2_). ESI(+)-MS: *m*/*z* 449 [M+Na]^+^, 427 [M+H]^+^. HREI-MS: *m*/*z* 411.12448 ± 0.0010, (M^+^˙− Me, calcd. for C_17_H_24_^35^ClN_4_O_4_Si: 411.12554), *m*/*z* 369.07799 ± 0.0010, (M^+^˙− *t*Bu, calcd for C_14_H_18_^35^ClN_4_O_4_Si: 369.07859).
4. *(3S,5S)-Methyl 3-(6-chloro-9H-purin-9-yl)-5-(hydroxymethyl)tetrahydrofuran-3-carboxylate (**5**).* Compound **4** (240 mg, 0.56 mmol) and tetrabutylammonium fluoride (TBAF, 2 M solution in THF, 1.35 mL, 0.676 mmol) were stirred for 2 h at room temperature. The crude product obtained by evaporation of the solvent was purified by FC using hexane/ethyl acetate gradient elution, to give pure **5**. TLC (dichloromethane/MeOH = 90:10): Rf 0.18. Yield: 87%. HPLC analysis (column CN, hexane/*i*-PrOH/MeOH 20:75:5) under UV detection at 254 nm showed a single peak at Rt = 18.2 min. [α]_D_^20^ = +18.2 (14.4 mg/mL, MeOH). FT-IR: 3400 (w, broad), 2900 (w broad), 1742 (s), 1590 (s), 1563 (s), 1436 (m), 1398 (m), 1343 (m), 1220 (s), 1050 (m), 934 (m), 834 (m), 636 (m). ^1^H NMR (400 MHz, CDCl_3_) δ 8.65 (s, 1H, H6′), 8.40 (s, 1H, H2′), 4.78 (d, *J* = 10.3 Hz, 1H, H_a_1) 4.46 (d, *J* = 10.3 Hz, 1H, H_b_1), 4.39 (m, 1H, H4), 3.68 (s, 3H, OMe), 3.81 (broad d, *J* = 12.1 Hz, 1H, H_a_ 5), 3.51 (dd, *J* = 12.1, 3.0 Hz, 1H, H_b_ 5), 3.10 (dd, *J* = 13.4, 8.0 Hz, 1H, H_a_ 3), 2.76 (dd, *J* = 13.4, 7.0 Hz, 1H, H_b_ 3). ^13^C NMR (100 MHz, CDCl_3_) δ 169.5 (COO), 151.9, 151.8 (C6′), 151.1, 144.2 (C2′), 132.0, 80.2 (C4), 74.5 (C2), 70.3 (C1), 62.7 (C5), 53.8 (OMe), 37.5 (C3). ESI(+)-MS: *m*/*z* 336 [M+Na]^+^, 313 [M+H]^+^. HRESI(+)-MS: *m*/*z* 313.07031 ± 0.0005, calcd. for C_12_H_14_^35^ClN_4_O_4_: 313.06981.
5. *(3S,5S)-Methyl 5-(hydroxymethyl)-3-(6-(4-methylpiperazin-1-yl)-9H-purin-9-yl)tetrahydrofuran-3-carboxylate (**6**).* Compound **5** (10 mg, 0.024 mmol) and *N*-methyl piperazine (0.008 mL, 0.072 mmol) in MeOH (1 mL) were microwave irradiated for 1.5 h at 70 °C. The crude mixture was purified by preparative TLC, using dichlorometane/MeOH = 85:15 *v*/*v* added of few drops of trimethylamine. TLC: (dichloromethane/MeOH = 80:20 *v*/*v*): Rf 0.63. Yield: 75%. HPLC analysis (column CN, hexane/*i*-PrOH/MeOH 20:75:5) under UV detection at 254 nm showed a single peak at Rt = 26.2 min. [α]_D_^20^ = +21.2 (6.66 mg/mL in MeOH). FT-IR: 3351 (w, broad), 2923 (w, broad), 1742 (m), 1693 (w), 1586 (s), 1453 (m), 1250 (m), 1046 (m), 793 (m), 646 (m). ^1^H NMR (400 MHz, CDCl_3_) δ 8.27 (s, 1H, H6′), 7.90 (s, 1H, H2′), 4.70 (d, *J* = 10.3 Hz, 1H, H_a_1) 4.53 (d, *J* = 10.3 Hz, 1H, H_b_1), 4.39 (m, 1H, H4), 4.31 (m, 4H), 3.70 (s, 3H, OMe), 3.80 (dd, *J* = 12.0, 2.6 Hz, 1H, H_a_ 5), 3.52 (dd, *J* = 12.0, 4.5 Hz, 1H, H_b_ 5), 3.09 (dd, *J* = 13.8, 7.5 Hz, 1H, H_a_ 3), 2.67 (dd, *J* = 13.8, 7.9 Hz, 1H, H_b_ 3), 2.54 (pseudo t, *J* = 4.6 Hz, 4H), 2.34 (s, 3H, NMe). ^13^C NMR (100 MHz, CDCl_3_) δ 170.2 (COO), 153.7, 152.3 (C6′), 151.2, 149.5, 136.5 (C2′), 79.9, 69.5, 63.2, 54.7, 53.4, 45.8, 45.6, 37.4 (C3). ESI(+)-MS: *m*/*z* 399 [M+Na]^+^, 377 [M+H]^+^; MS/MS (377): *m*/*z* 320, 162. HRESI(+)-MS: *m*/*z* 377.19344 ± 0.0003, [M+H^+^], calcd. for C_17_H_25_N_6_O_4_: 377.19318).
6. *(3S,5S)-Methyl 5-(hydroxymethyl)-3-(6-(methylamino)-9H-purin-9-yl)tetrahydrofuran-3-carboxylate (**7**).* Compound **5** (25 mg, 0.081 mmol) and methylamine (2M in THF, 0.230 mL, 0.46 mmol) in MeOH (0.5 mL) were microwave irradiated for 20 min at 70 °C. The crude mixture was purified by preparative TLC was purified by preparative TLC, using dichloromethane/MeOH = 90:10 *v*/*v* added of few drops of trimethylamine, obtaining pure **7** as a light-yellow solid. TLC (dichloromethane/MeOH = 90:10 *v*/*v*). Rf 0.25. Yield 92%. [α]_D_^20^= +16.9 (12.5 mg/mL in MeOH). FT-IR (cm^−1^): 3364 broad w, 2925 m, 1740 m, 1623 s, 1456 w, 1376 w, 1297 w, 1241 m, 1045 m, 963 w, 645 s broad. ^1^H NMR (400 MHz, CDCl_3_) δ 8.25 (s, 1H, H6′), 7.97 (s, 1H, H2′), 6.28 (br s, 1H), 4.85 (d, *J* = 10.3 Hz, 1H, H_a_1) 4.43 (d, *J* = 10.3 Hz, 1H, H_b_1), 4.37 (m, 1H, H4), 3.65 (s, 3H, OMe), 3.83 (brd, *J* = 11.7 Hz, 1H, H_a_ 5), 3.51 (dd, *J* = 11.7, 3.6 Hz, 1H, H_b_ 5), 3.26 (m, 1H), 3.05 (br s, 3H, NMe), 2.98 (dd, *J* = 13.6, 8.1 Hz, 1H, H_a_ 3), 2.83 (dd, *J* = 13.6, 6.5 Hz, 1H, H_b_ 3), 3.05 (br s, 3H, NMe). ^13^C NMR (100 MHz, CDCl_3_) δ 170.3 (COO), 155.1, 153.2 (C6′), 148.8, 138.4 (C2′), 119.7, 80.5, 74.5, 69.8 (C1), 62.6 (C5), 53.4 (OMe), 37.2 (C3), 27.2 (NMe). ESI(+)-MS: *m*/*z* 308 [M+H]^+^, MS/MS (308): *m*/*z* 290, 248; HRESI(+)-MS: *m*/*z* 308.13566 ± 0.0004, [M+H]^+^, calcd. for C_13_H_18_N_5_O_4_: 308.13533.
7. *(3S,5S)-Isopropyl 3-(6-chloro-9H-purin-9-yl)-5-(hydroxymethyl)tetrahydrofuran-3-carboxylate (**8**).* Compound **5** (30 mg, 0.08 mmol,) and triazabicyclodecene (TBD, 3 mg, 0.024 mmol) in *i*-PrOH (1 mL) were microwave irradiated for 30 min at 50 °C. The crude mixture was purified using CN-column chromatography using dichloromethane/MeOH gradient elution to obtain pure **8** as light-yellow solid. TLC: CN phase, dichloromethane/MeOH 99:1 *v*/*v*. Rf = 0.56. Yield 53%. [α]_D_^20^ = +26.1 (17.4 mg/mL in MeOH). FT-IR (cm^−1^): 3400 (m, broad), 2925 (m), 1720 (s), 1688 (s), 1589 (m), 1453 (m), 1376 (m), 1228 (s), 1100 (s), 1039 (s), 827 (m), 603 (s). ^1^H NMR (400 MHz, CDCl_3_) δ 8.68 (s, 1H, H6′), 8.26 (s, 1H, H2′), 4.76 (d, *J* = 10.3 Hz, 1H, _Ha1_) 4.42 (d, *J* = 10.3 Hz, 1H, H_b_1), 5.00 (sept, *J* = 6.4 Hz, 1H, O*i*Pr), 4.37 (m, 1H, H4), 3.80 (dd, *J* = 12.5, 2.5 Hz, 1H, H_a_ 5), 3.50 (dd, *J* = 12.5, 3.8 Hz, 1H, H_b_ 5), 3.07 (dd, *J* = 13.6, 8.0 Hz, 1H, H_a_ 3), 2.72 (dd, *J* = 13.6, 7.2 Hz, 1H, H_b_ 3), 2.97 (br s, 1H), 1.06 (pseudo t, *J* = 6.4Hz, 6H, *i*-Pr). ^13^C NMR (100 MHz, CDCl_3_) δ 168.2 (COO), 151.9, 151.0, 151.6 (C6′), 144.1 (C2′), 131.8, 80.2 (C4), 74.4 (C2), 71.2 (C1), 70.6 (*i*-PrO), 62.7 (C5), 37.4 (C3), 21.4 (*i*-PrO). ESI(+)-MS: *m*/*z* 341 [M+H]^+^, 323 [M+H-H_2_O]^+^. HRESI(+)-MS: *m*/*z* 341.10181 ± 0.0007, calcd. for C_14_H_18_^35^ClN_4_O_4_: 341.10111.
8. *(3S,5S)-Methyl 3-(6-amino-9H-purin-9-yl)-5-(hydroxymethyl)tetrahydrofuran-3-carboxylate (**9**).* Diethylazodicarboxylate (DEAD, 40% in toluene, 721 mg, 4.14 mmol) was slowly added to triphenylphosphine (1084 mg, 4.14 mmol) under N_2_ atmosphere at 0 °C. After 30 min, **3** (300 mg, 1.03 mmol) and adenine in THF were added, stirring under reflux for 10 h, monitoring by TLC (hexane/EtOAc 6:4 *v*/*v* with few drops of trimethylamine). The reaction mixture was concentrated and the residue subjected to FC using hexane/AcOEt added of 1% triethylamine gradient elution. The recovered product was then treated with TBAF in THF stirring 2 h at room temperature as described in the procedure for obtaining **5**. The deprotected product was purified using CN-column chromatography with hexane/AcOEt gradient elution obtaining pure **9** as light yellow solid TLC: dichloromethane/MeOH 85:15 *v*/*v*. Rf = 0.32. Yield 52%. [α]_D_^20^ = +16.8 (8.57 mg/mL in MeOH). FT-IR (cm^−1^) 3193 (m, broad), 2928 (m), 1704 (m), 1642 (m), 1581 (s), 1437 (s), 1250 (s), 1119 (vs), 837 (m). ^1^H NMR (400 MHz, CDCl_3_) δ 8.19 (s, 1H, H6′), 8.03 (s, 1H, H2′), 6.06 (br s, 2H), 4.84 (d, *J* = 10.3 Hz, 1H, H_a_1) 4.45 (d, *J* = 10.3 Hz, 1H, H_b_1), 4.41 (m, 1H, H4), 3.69 (s, 3H, OMe), 3.87 (dd, *J* = 12.6, 2.6 Hz, 1H, H_a_ 5), 3.53 (dd, *J* = 12.6, 3.6 Hz, 1H, H_b_ 5), 3.05 (dd, *J* = 13.8, 8.1 Hz, 1H, H_a_ 3), 2.84 (dd, *J* = 13.8, 7.2 Hz, 1H, H_b_3). ^13^C NMR (100 MHz, CDCl_3_) δ 170.0 (COO), 155.3, 152.7 (C6′), 150.0, 139.0 (C2′), 119.6, 80.4, 74.6, 69.8 (C1), 62.6 (C5), 53.5 (OMe), 37.3 (C3). EI-MS: *m*/*z* 293 [M^+.^]; HRESI(+)-MS: *m*/*z* 294.11985 ± 0.0003, [M+H] ^+^, calcd. for C_12_H_15_N_5_O_4_: 294.11968.
9. *(3S,5S)-Isopropyl 3-(6-amino-9H-purin-9-yl)-5-(hydroxymethyl)tetrahydrofuran-3-carboxylate (**10**).* Compound **9** (30 mg, 0.10 mmol) and TBD (4.3 mg, 0.03 mmol) *i*-PrOH (1 mL) were microwave irradiated for 30 min at 50 °C. The crude product was purified by FC using dichloromethane/MeOH gradient elution, to obtain **10** as a pure white solid. TLC: dichloromethane/MeOH 90:10 *v*/*v*, +1% triethylamine. Rf = 0.57. Yield: 51%. [α]_D_^20^ = +13.0 (9.4 mg/mL in MeOH). FT-IR (cm^−1^) 3333 (m, broad), 2926 (m), 2360 (w), 1734 (m), 1641 (s), 1600 (s), 1473 (m), 1292 (m), 1226 (s), 1102 (s), 1042 (m), 799 (m), 647 (m). ^1^H NMR (400 MHz, CDCl_3_) δ 8.21 (s, 1H), 8.03 (s, 1H), 5.93 (br s, 2H, NH_2_), 4.81 (d, *J* = 10.2 Hz, 1H, H_a_1) 4.45 (d, *J* = 10.2 Hz, 1H, H_b_1), 5.03 (sept, *J* = 6.1 Hz, 1H, *i*-PrO), 4.40 (m, 1H, H4), 3.87 (brd, *J* = 12.5 Hz, 1H, H_a_5), 3.54 (dd, *J* = 12.5, 3.9 Hz, 1H, H_b_5), 3.04 (dd, *J* = 13.7, 8.1 Hz, 1H, H_a_3), 2.80 (dd, *J* = 13.7, 7.6 Hz, 1H, H_b_3), 1.08 ( d, *J* = 6.1Hz, 6H, *i*-Pr). ^13^C NMR (100 MHz, CDCl_3_) δ 168.9 (COO), 155.3, 152.6, 150.1, 139.0, 119.5, 80.4, 74.5, 70.7, 70.0, 62.7, 37.1, 21.3. ESI(+)-MS: *m*/*z* 344 [M+Na]^+^, 322 [M+H]^+^; HRESI(+)-MS: *m*/*z* 322.15094 ± 0.0003, [M+H]^+^, calcd. for C_14_H_20_N_5_O_4_: 322.15098.
10. *(3S,5S)-3-(6-Amino-9H-purin-9-yl)-5-(hydroxymethyl)-N-methyltetrahydrofuran-3-carboxamide (**11**).* Methylamine (2M in THF, 0.273 mmol, 0.137 mL) was slowly added to a solution of **9** (20 mg, 0.68 mmol) in MeOH (1 mL) at 0 °C. The reaction was stirred overnight at r.t., then concentrated in vacuo and the residue subjected to FC, using dichloromethane/MeOH gradient elution, to obtain pure **11** as white solid. TLC: dichloromethane/MeOH 85:15 *v*/*v*. Rf: 0.23. Yield: 60%. [α]_D_^20^= +21.4 (7.8 g/mL in MeOH). FT-IR (cm^−1^): 3326 m broad, 2926 w, 1639 s, 1601 m, 1573 m, 1475 m, 1414 m, 1376 m, 1339 m, 1260 m, 1226 m, 1042 m, 644 s broad. ^1^H NMR (400 MHz, acetone-d_6_) δ 8.16 (s, 1H), 8.14 (s, 1H), 6.65 (br s, 2H, NH_2_), 4.65 (d, *J* = 9.8 Hz, 1H, H_a_1) 4.56 (d, *J* = 9.8 Hz, 1H, H_b_1), 4.29 (m, 1H, H4), 3.50 (dd, *J* = 11.6, 4.2 Hz, 1H, H_a_ 5), 3.46 (dd, *J* = 11.6, 4.8 Hz, 1H, H_b_ 5), 3.14 (dd, *J* = 13.4, 7.8 Hz, 1H, H_a_ 3), 2.67 (dd, *J* = 13.4, 7.6 Hz, 1H, H_b_3), 2.66 (d, *J* = 4.6 Hz, 3H, NMe). ^13^C NMR (100 MHz, acetone-d_6_) δ 169.4 (COO), 156.2, 152.5 (C6′), 150.2, 139.6 (C2′), 120.1, 79.8, 73.9, 70.3 (C1), 63.3 (C5), 37.5 (C3), 25.7 (NMe). ESI(+)-MS *m*/*z* 293 [M+H]^+^; MS/MS(293): *m*/*z* 234; HRESI(+)-MS: *m*/*z* 293.13586±0.0003, [M+H]^+^, calcd. for C_12_H_17_N_6_O_3_: 293.13566.

### 3.2. Computational Analysis

#### 3.2.1. ADME Predictions

ADME prediction were performed using the Online Server Swiss-ADME [19].

#### 3.2.2. Docking Calculation

Calculations were carried out on a PC running at 3.4 GHz on an Intel i7 2600 quad core processor with 8 GB RAM and 1 TB hard disk with Windows 7 Home Premium 64-bit SP1 as an operating system. Ligands were built and minimized using MMX force field by PC Model version 9.3 (Serena Software, Bloomington, IN, USA). The minimized molecules were saved in pdb extension. The structures of RNA-dependent RNA polymerase from SARS-CoV-2 bound to the template-primer RNA and phosphate form of remdesivir (PDB ID: 7BV2) was determined by electron microscopy with a resolution of 2.5 Å [13]. The structures were modified as follows: all the crystallization water molecules and the ions were removed; the Protein—template-primer RNA containing in its structure remdesivir was separated in protein and nucleic acid molecule respectively and saved in pdb extension. All hydrogen atoms as well as atomic charge were added to both structures and minimized by using Yasara 2 force field. The structure of nucleic acid was modified by substitution the residue of remdesivir with each molecule under investigation and the obtained structure was minimized with the same force field and saved in pdb extension. The obtained structures were submitted to PatchDock Server [20] by using the default clustering RMSD at 4 Å [14,21]. The results were reported as geometric shape complementarity score values, Interface area of the complexes values and as pdb structures complexes. The pdb result complexes were further minimized with the same force field to optimize the interactions between the macromolecules. The visual interactions in ligand–RNA–enzyme were displayed using Discovery Studio Visualizer v.19.1.0.18287 [22].

#### 3.2.3. Molecular Dynamics Procedure

Calculations were carried out on a PC running at maximum clock of 4.9 GHz on an AMD-Ryzen9 5950×, 16 core processor (32 threads) with 32 GB RAM and 1 TB hard disk with Windows 10 at 64-bit as an operating system by using Yasara software (YASARA Biosciences GmBH, Vienna, Austria) [23] The structure of complex, obtained from PatchDock server calculation and saved in pdb file format, was cleaned, added of hydrogen atoms and hydrogen bond network optimized. A cubic cell was constructed with a periodic boundary condition. The extension of the cell on each side around the solute was 10 Å, meaning that the cell will be 20 Å larger than the protein. The force field used was YASARA2, which have been optimized for structure prediction, refinement, energy minimization and validation. The ligand was parameterized by AutoSMILES [24] algorithm, which use a combination of AM1BCC [25] and General AMBER Force Field (GAFF) [26] for typing atomic charges and bond orders in organic molecules. The TIP3P solvation system was used with 0.997 g/L density for solvating the simulation box [27] and protonation state of titratable amino acids in the protein were predicted. The pH of the system was maintained at 7.4 to mimic physiological conditions. After addition of Na^+^ and Cl^−^ ions at final concentration of 0.9% (m/V) [28], a steepest descent energy minimization, followed from simulated annealing protocol was applied to reduce the conformational stress of the system. In order to explain the long-range electrostatic interactions, the Ewald particle mesh (PME) was used, setting the distance cut-off at 8 Å. The molecular dynamic simulation was performed for 35 ns by using the Berendsen thermostat at 298 K, with a time step interval of 2.5 fs together with a multiple time step algorithm [29,30] The pressure was set to constant, and trajectories were accumulated with an interval of 100 ps [29,30] The intermolecular force calculation was saved every two simulation sub-steps whereas simulation snapshots every 100 ps.

The system composition for compound **6** simulation resulted in 16,104 atoms for the protein, 790 atoms in nucleic acid, 54 atoms in ligand **6**, 177 Na^+^ ions, 149 Cl^−^ ions and 57,093 molecules of water for a total of 188,553 atoms in the soup. The system composition for GS-441524 simulation resulted in 16,104 atoms in the protein, 790 atoms in nucleic acid, 36 atoms in GS-441524 ligand, 177 Na^+^ ions, 150 Cl^−^ ions and 57,504 molecules of water for a total of 189,771 atoms in the soup.

#### 3.2.4. Minimized Structure of Compound **5** by DFT Calculation

DFT calculation was performed in the gas phase and in chloroform by using Polarized Continuum Model (PCM) [31] Calculations were carried out on a PC running at 3.4 GHz on an Intel i7 2600 quad core processor with 8 GB RAM and 1 TB hard disk with Windows 7 Home Premium 64-bit SP1 as an operating system. Structure of compound **5** was built using PC Model version 9.3 (Serena Software, Bloomington, IN, USA) and minimized by MM using MMX force field. Conformational analysis was achieved by GMMX search module present in PC Model. Gaussian 03W revision E.01 program [32] with graphical interface GaussView 4.0 was used in the geometry optimization at a density functional theory (DFT) level of theory. The optimized geometry was obtained by using RFO step and type convergence criteria and invoking gradient employing a mixed basis set as following: 6-31G(d,p) for hydrogen, carbon, nitrogen and oxygen atoms, and 6-311G(d) for chlorine atom. The electronic correlation functional B1B95, where the gradient-corrected DFT with Becke hybrid functional B1 [33] for the exchange part and the B95 for correlation function [34,35,36] was utilized. The optimized structural parameters were taken in the vibrational energy calculations at the DFT levels to characterize all stationary points as minima. No imaginary wave number modes were obtained for the optimized structure, proving that a local minimum on the potential energy surface was actually found.

## 4. Conclusions

A series of new enantiomerically pure nucleoside analogues were synthesized from the chiral building block LAC obtained as one added value product from cellulose pyrolysis in the context of biorefinery. By a convergent synthetic approach, seven molecules showing modifications on the nucleobase and on the carboxyl moiety on the tetrahydrofurane ring have been obtained through a Mitsunobu substitution where NOE experiments proved the configurational inversion supported by energy-minimized structure DFT calculation. ADME and drug-likeness prediction showed favorable parameters in comparison with the recently approved anti COVID-19 agent remdesivir and its metabolite GS-441524. Docking calculation took into account the complex system formed by RdRp enzyme and each small molecule covalently linked to primer RNA. For compound **6**, emerging as the most promising structure in terms of pharmacokinetic parameters and by docking calculation, MD simulation showed a stable molecular interaction in RdRp complex and a minor average atomic fluctuation (RMSF) than remdesivir metabolite, suggesting a well accommodation in the RdRp pocket.

In summary, the whole computational study provided data also better than remdesivir metabolite, all agree in indicating compound **6** as a promising candidate to deepen the study on a potential therapeutic inhibitor of SARS-CoV-2 RNA-dependent RNA polymerase. These findings emphasize the interest to deepen the study in next biological evaluation.

## Figures and Tables

**Table 1:** Geometric shape complementarity score and approximate interface area of the complex for each indicated compounds with free RdRp (7BV2) by computational analysis described in Figure 6.

Compound	Remdesivir	5	6	7	8	9	10	11
Score	16,264	17,496	19,052	18,336	17,460	18,538	18,118	17,918
Area (Å^2^)	1924	2477	2362	2257	2302	2257	2093	2295

**Table 2:** Interactions (H-bond, π-π or π-alkyl, van der Waals, charge–charge or π-on and unfavorable) and in brackets the relative distance in Å evaluated by docking calculations of compounds **5**–**11** and remdesivir metabolite, each of them linked to RNA in the receptor pocket of free RdRp (7BV2).

Compound	H-Bond	π-π π-Alkyl	vdW	ch-ch π-ion	Unf.
GS-441524	U10 (3.07, 2.90) U20 (2.93, 2.99) Asn691 (2.73) Ser759 (2.30) Asp760 (1.40)	A11 (3.66, 5.04) U20 (4.17, 5.38)	Ala688 (2.76) Ser759 (2.62)	Lys551 (5.05), Asp760 (4.79)	Cys622 (2.42) Asp760 (3.79)
5	Arg555 (1.79, 2.77) Ser759 (1.71) Asp760 (1.10)	U10 (4.13, 5.94)	U10 (4.11) Lys545 (4.55) Val557 (4.41) Ser682 (2.55, 2.77)	Asp760 (3.21)	-
6	U20 (1.73) Ile548 (1.83) Arg553(2.69) Arg555 (2.40) Ser759 (2.41) Asp760 (1.40)	-	U10 (5.17), A11 (4.75) Ala547 (2.22), Ser682 (2.62)	Arg555 (2.97, 3.61)	-
7	Arg555 (1.77) Thr556 (2.07) Asp760 (1.14)	Ser681 (3.69, 4.69)	Thr556 (2.76) Thr687 (2.27) Ser759 (2.71) Asp760 (2.81)	Ser682 (4.77), Asp760 (3.15)	-
8	Lys551 (1.77) Ser759 (1.61)	U10 (4.80) A11 (4.75)	U20 (2.39) Ala688 (3.81) Asp760 (2.67) Ser814 (2.89)	Lys551 (1.67)	Lys551 (3.03)
9	U20 (1.59) Arg553 (1.83) Arg555 (1.88) Asp760 (1.10)	-	Thr556 (2.47) Val557 (4.59) Cys622 (5.21) Asp62 (2.45) Ser682 (2.79) Asp760 (2.32)	Asp760 (3.09)	-
10	U20 (2.22) Arg555 (1.93, 2.21)	-	A11 (4.91), Leu758 (2.66) Asp760 (2.29)	Asp760 (4.82) Asp761(4.43)	Asp760 (4.99)
11	A11(2.56) U20 (1.68) Arg553 (2.24) Asp760 (1.10)	U20 (5.42)	Arg555 (5.31) Asp760 (2.53) Asp763 (2.97) Ser682 (2.37, 2.51)	Arg555 (4.92, 4.96) Asp760 (3.11)	Cys622 (2.11)

## Data Availability

The data presented in this study are available on request from the corresponding author.

## Supplementary Materials

The following supporting information can be downloaded at: https://www.mdpi.com/article/10.3390/ijms23010518/s1.
