# Supplementary material for: Synthesis of Nucleoside-like Molecules from a Pyrolysis Product of Cellulose and Their Computational Prediction as Potential SARS-CoV-2 RNA-Dependent RNA Polymerase Inhibitors
Source: Int J Mol Sci. 2022 Jan 4;23(1):518. doi: 10.3390/ijms23010518 (PMC8745730; doi:10.3390/ijms23010518)
Supplement: Supplementary file 1 [file ijms-23-00518-s001.zip › ijms-1530382-supplementary.pdf]

Supplementary Material

Synthesis of nucleoside-like molecules from a pyrolysis product of cellulose and their computational prediction as SARS-CoV-2 RNA-dependent RNA polymerase inhibitors

Andrea Defant [1,*], Federico Dosi [1] , Nicole Innocenti [1] and Ines Mancini [1,*]

[1] Università di Trento, Dipartimento di Fisica, Laboratorio di Chimica Bioorganica, via Sommarive 14, 38123, Povo, Trento, Italy

* Correspondence: ines.mancini@unitn.it ; Tel.: +39-0461-281-548; andrea.defant@unitn.it

Table of Contents

Figure S1. ^{1}HNMR (400MHz) and ^{13}CNMR (100MHz) spectra of compound **5** in $CDCl_3$.

Figure S2. ^{1}HNMR (400MHz) and ^{13}CNMR (100MHz) spectra of compound **6** in $CDCl_3$.

Figure S3. ^{1}HNMR (400MHz) and ^{13}CNMR (100MHz) spectra of compound **7** in $CDCl_3$.

Figure S4. ^{1}HNMR (400MHz) and ^{13}CNMR (100MHz) spectra of compound **8** in $CDCl_3$.

Figure S5. ^{1}HNMR (400MHz) and ^{13}CNMR (100MHz) spectra of compound **9** in $CDCl_3$.

Figure S6. ^{1}HNMR (400MHz) and ^{13}CNMR (100MHz) spectra of compound **10** in $CDCl_3$.

Figure S7. ^{1}HNMR (400MHz) and ^{13}CNMR (100MHz) spectra of compound **11** in acetone-d_6.

Figure S8. Chemical structure and bioavailability radar for compounds **5-11**, remdesivir and GS-441524 evaluated by Swiss-ADME.

Figure S9. Drug-likeness prediction by Molsoft server (https://molsoft.com/mprop/)

Figure S10. Two-dimensional representations for the interactions of compounds **5** and **7-11** linked to RNA in the receptor pocket of free RdRp (7BV2), as deduced by docking calculation.

Figure S11. Data from MD simulation: (a) total potential energy of the system during the time and (b) quality Z-score of structure for free RdRp on the left, compound **6** at the center and GS-441524 on the right.

Figure S12. Data from MD simulation: (a) radius of gyration (in Å) during all simulation time and (b) RMSD (in Å) from the starting structure (Cα in blu, backbone in red and all heavy atoms in green) for free RdRp on the left, compound **6** at the center and GS-441524 on the right.

Table S1. ADME Prediction of compounds **5-11** and reference compounds remdesivir and GS-441524 evaluated by on-line Server Swiss-ADME (http://www.swissadme.ch/).

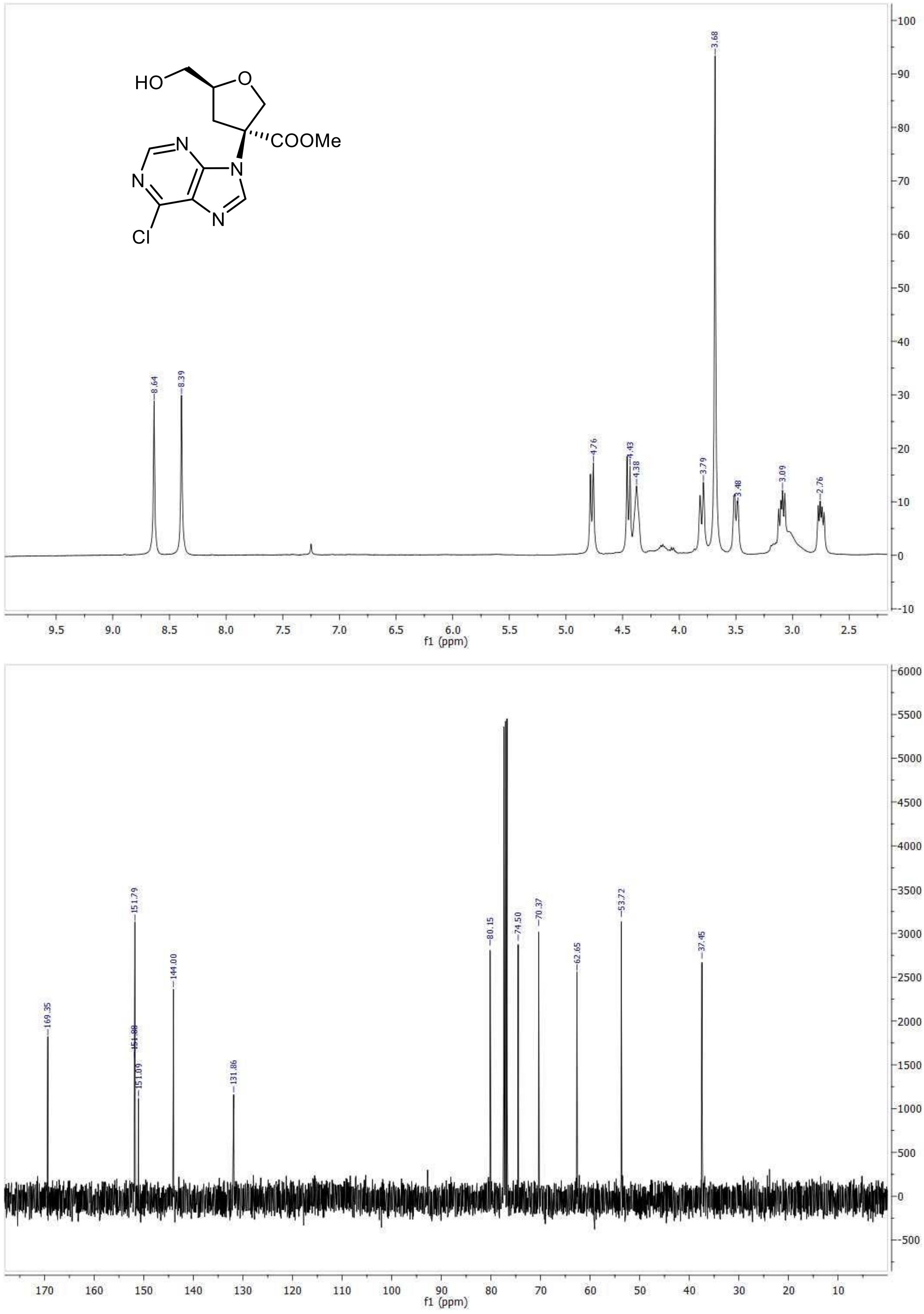

Figure S1. ^{1}HNMR (400MHz) and ^{13}CNMR (100MHz) spectra of compound **5** in $CDCl_3$.

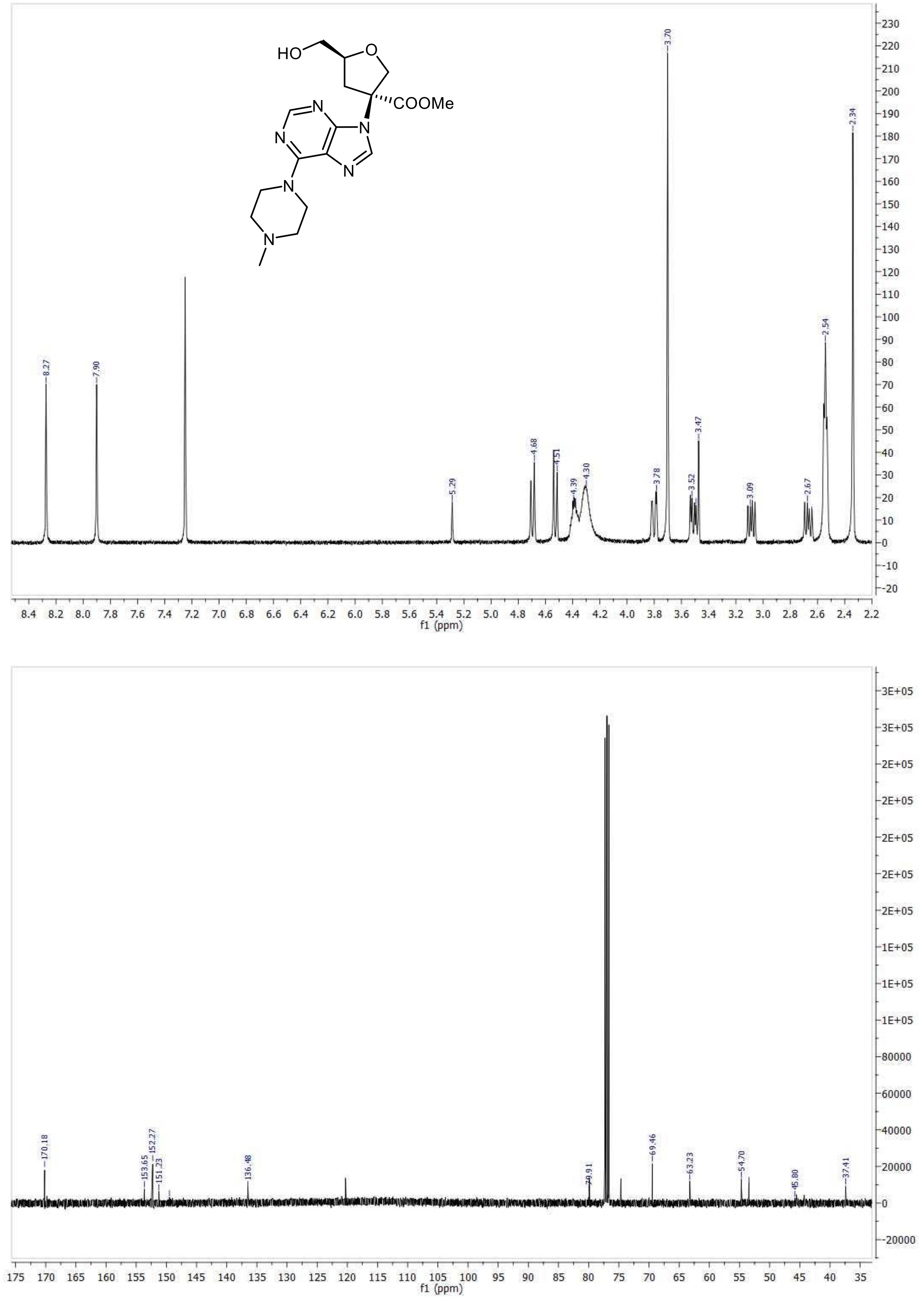

Figure S2. ^{1}HNMR (400MHz) and ^{13}CNMR (100MHz) spectra of compound **6** in $CDCl_3$.

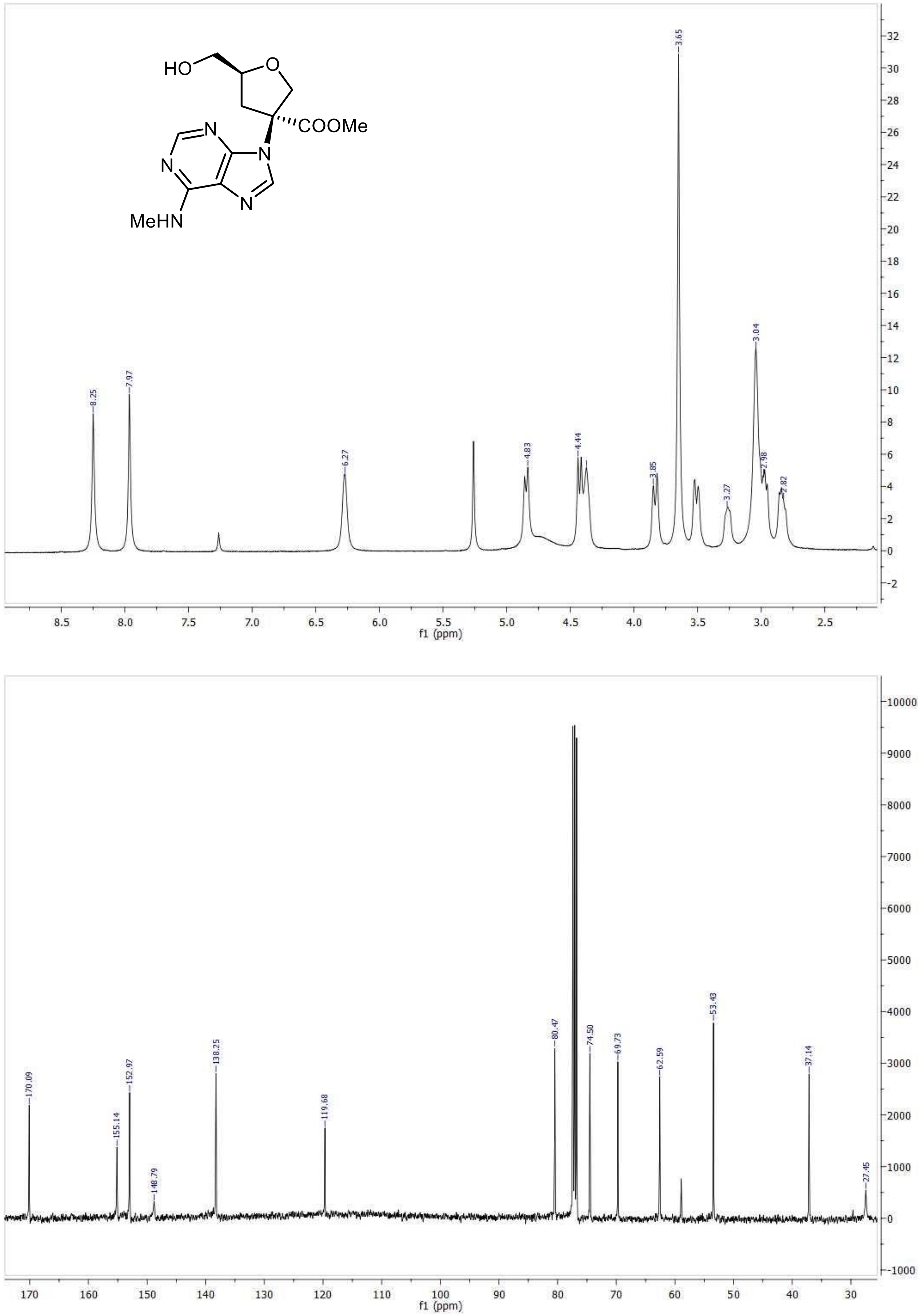

Figure S3. ^{1}HNMR (400MHz) and ^{13}CNMR (100MHz) spectra of compound **7** in $CDCl_3$.

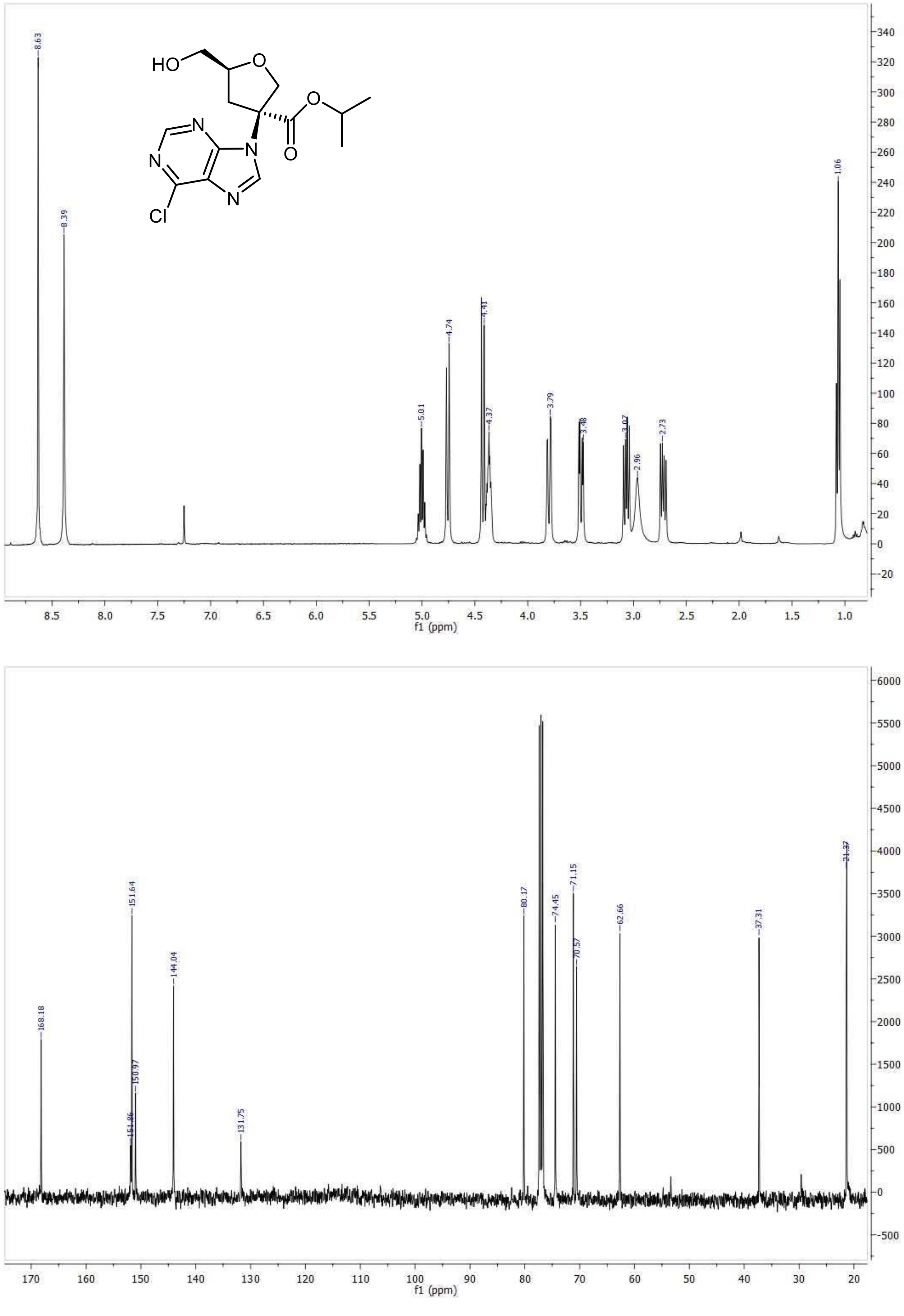

Figure S4. ^{1}HNMR (400MHz) and ^{13}CNMR (100MHz) spectra of compound **8** in $CDCl_3$.

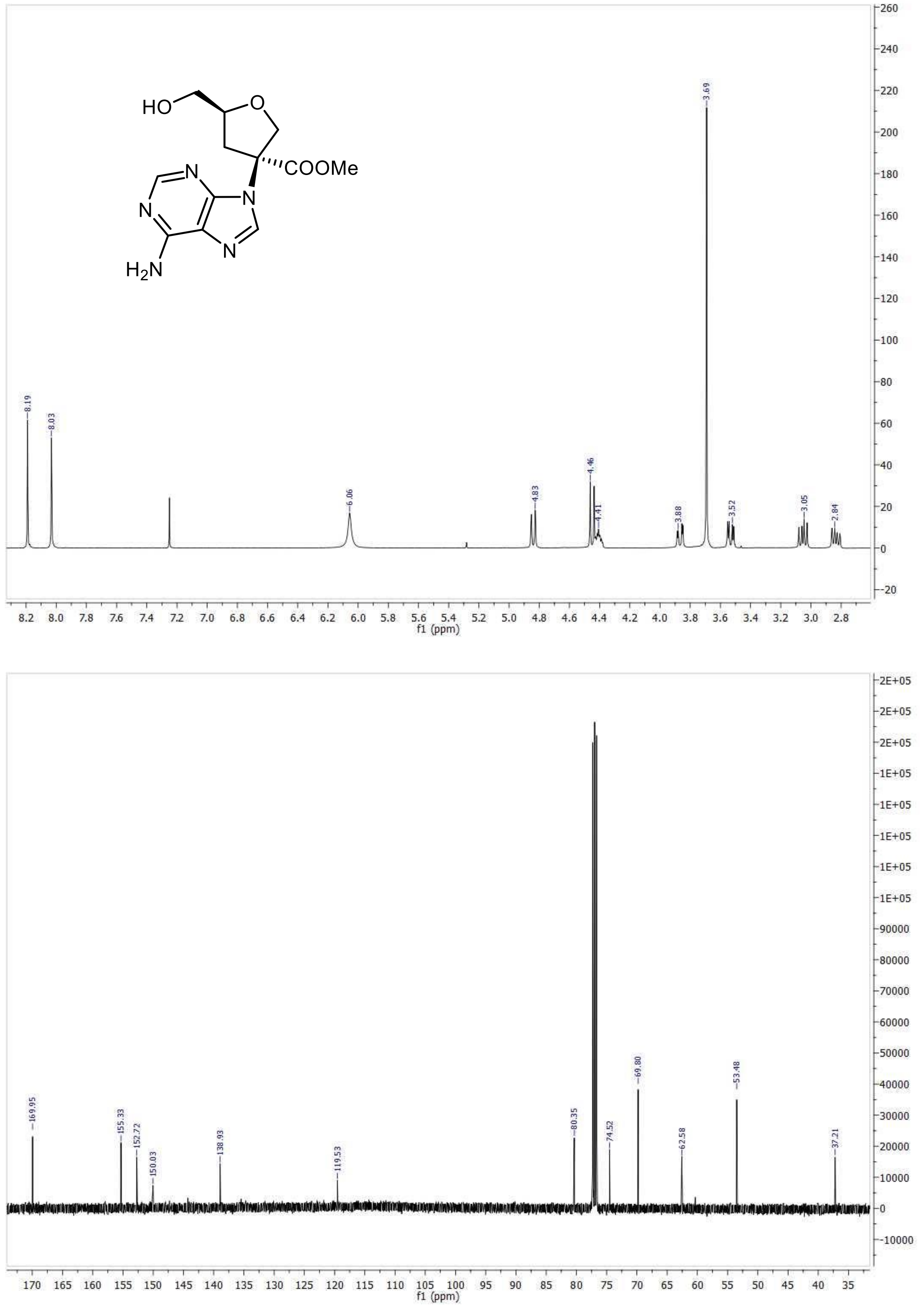

Figure S5. ^{1}HNMR (400MHz) and ^{13}CNMR (100MHz) spectra of compound **9** in $CDCl_3$.

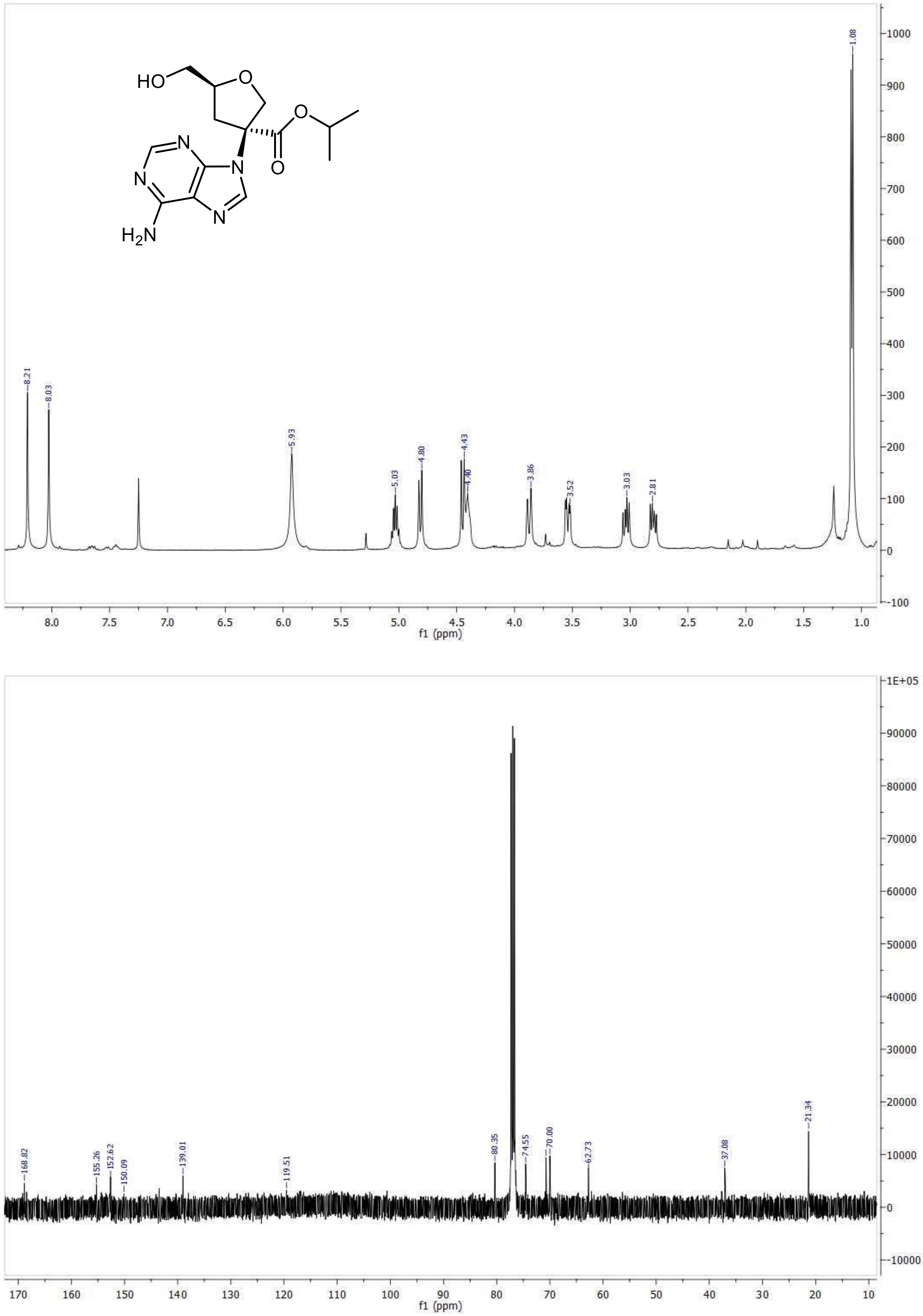

Figure S6. ^{1}HNMR (400MHz) and ^{13}CNMR (100MHz) spectra of compound **10** in $CDCl_3$

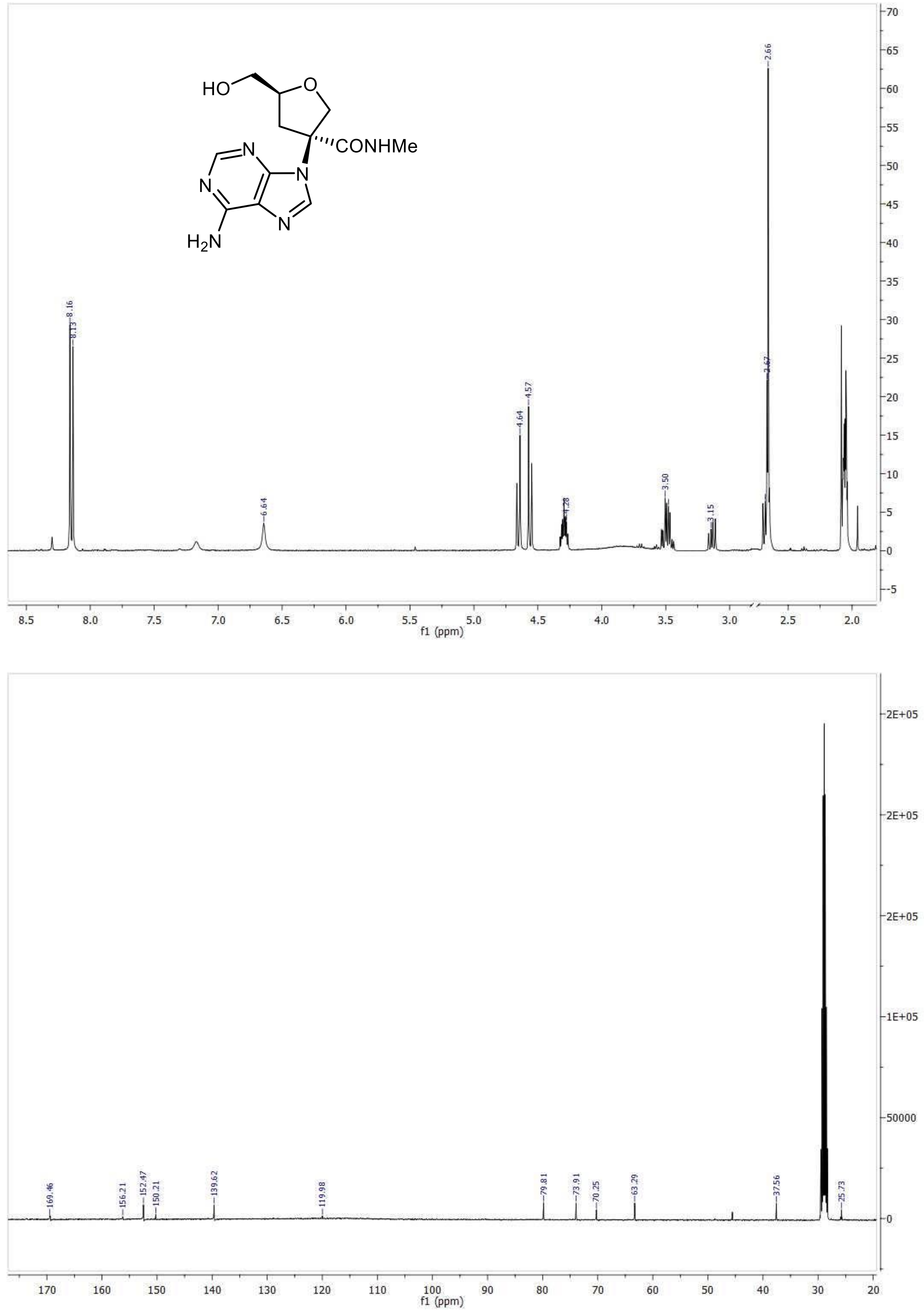

Figure S7. ^{1}HNMR (400MHz) and ^{13}CNMR (100MHz) spectra of compound **11** in acetone-d6

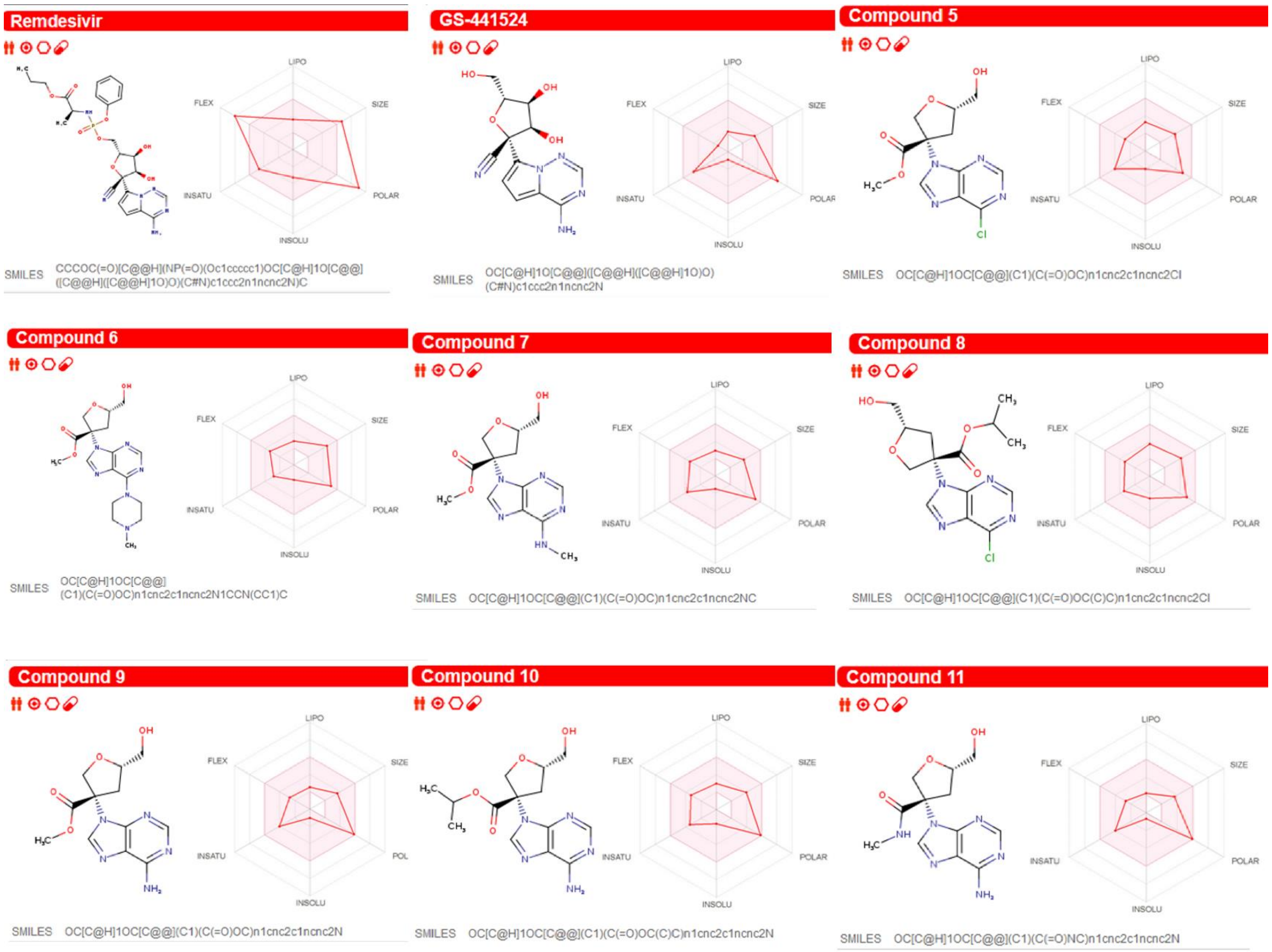

Figure S8. Chemical structure and bioavailability radar for compounds **5**-**11**, remdesivir and GS-441524 evaluated by Swiss-ADME.

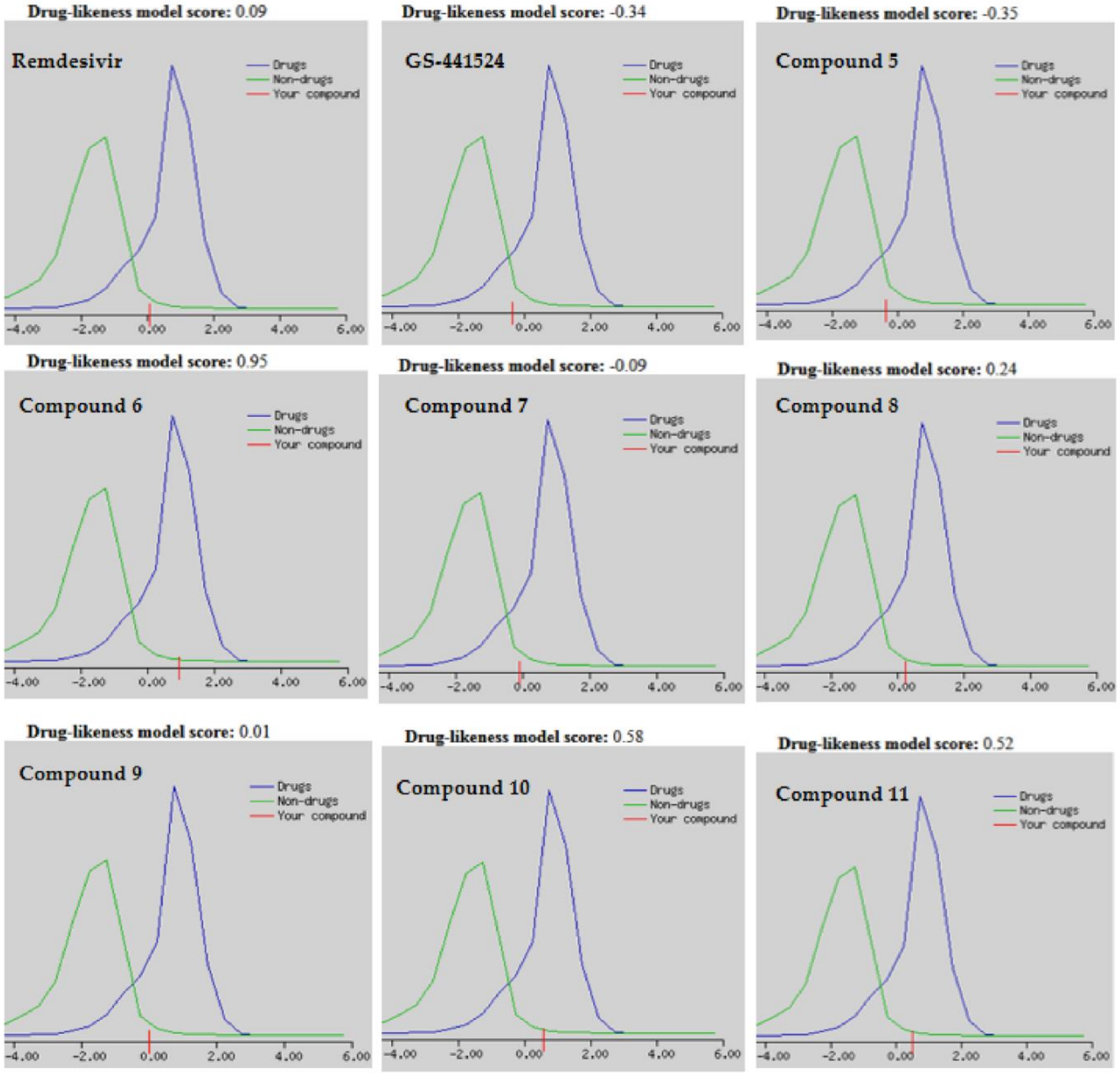

Figure S9. Drug-likeness prediction for compounds **5-11**, remdesivir and GS-441524 by Molsoft server (https://molsoft.com/mprop/)

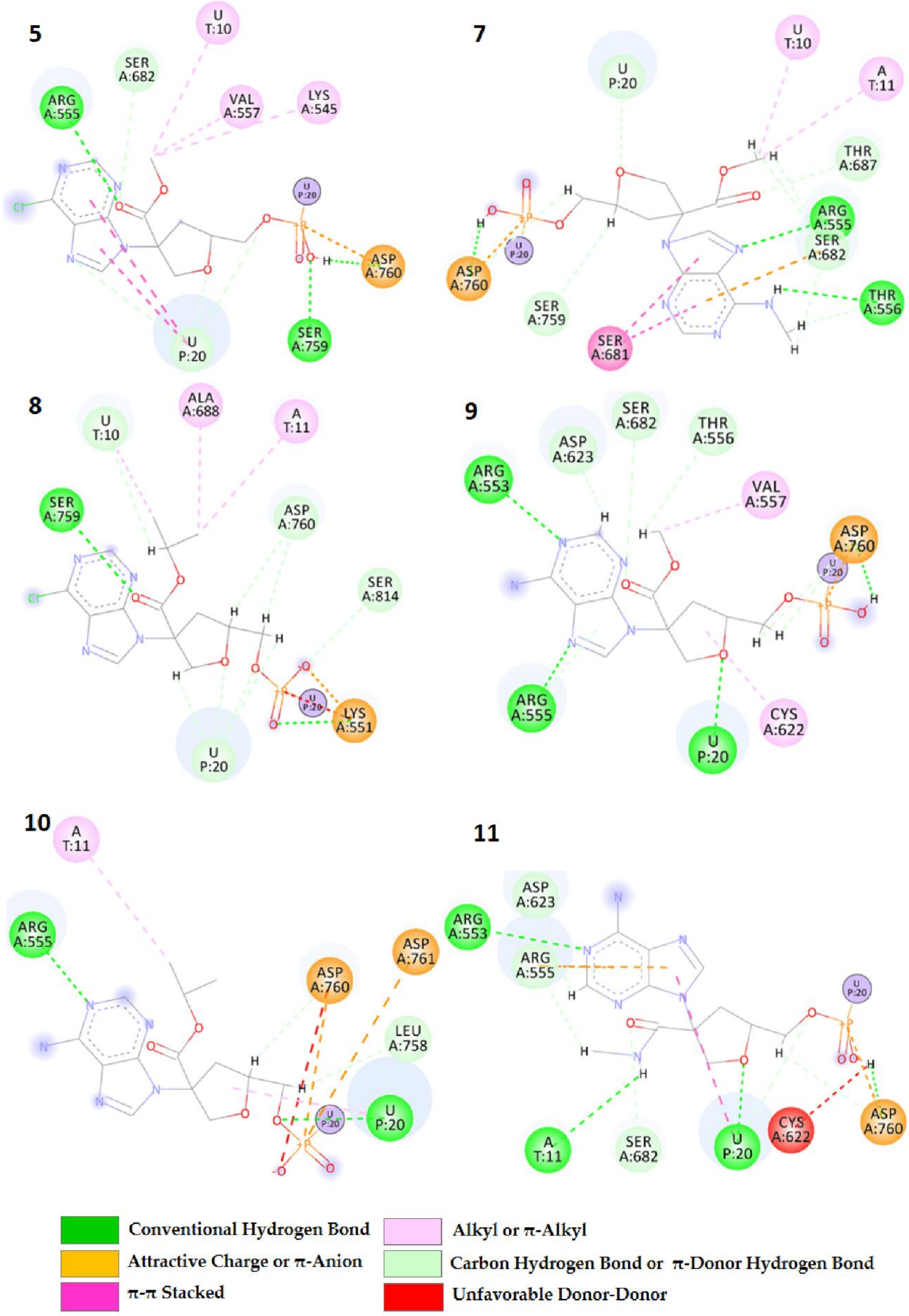

Figure S10. Two-dimensional representations for the interactions of compounds **5** and **7-11** linked to RNA in the receptor pocket of free RdRp (7BV2), as deduced by docking calculation.

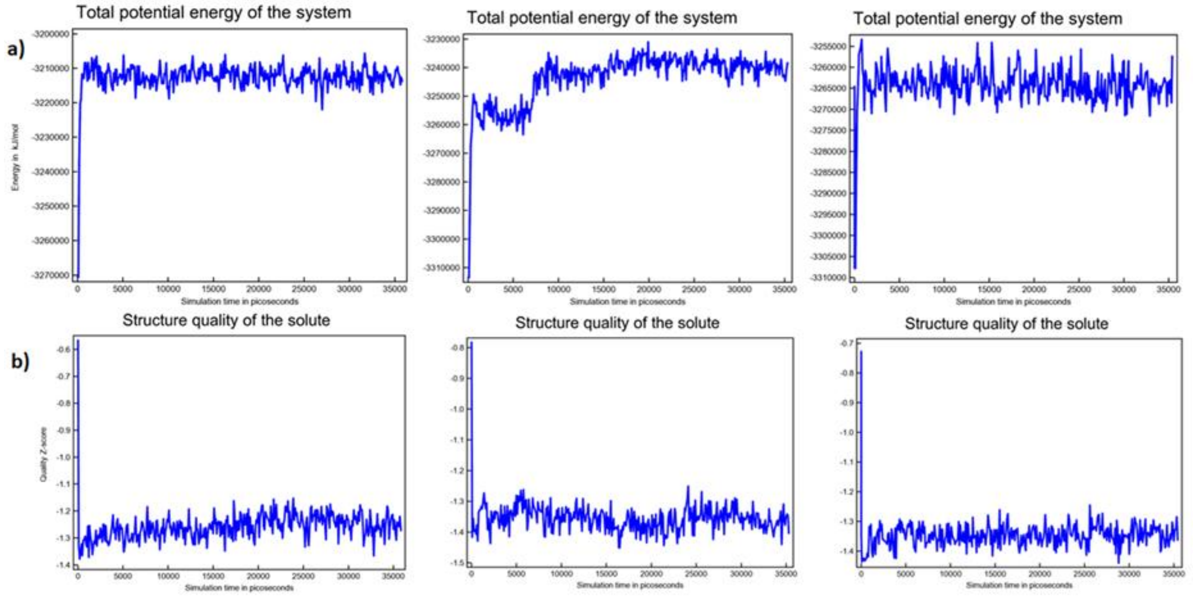

Figure S11. Data from MD simulation: (a) total potential energy of the system during all simulation time and (b) quality Z-score of structure for free RdRp on the left, compound **6** at the center and GS-441524 on the right.

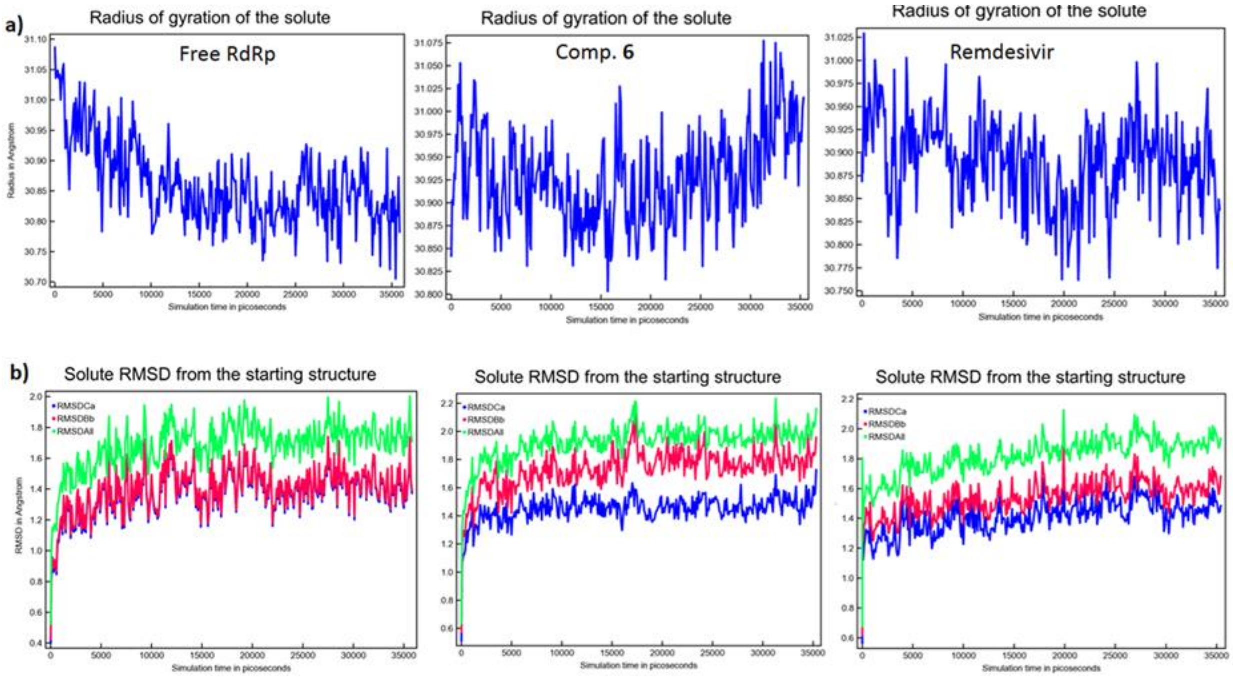

Figure S12. Data from MD simulatio: (a) radius of gyration (in Å) during all simulation time and (b) RMSD (in Å) from the starting structure (Cα in blu, backbone in red and all heavy atoms in green) for free RdRp on the left, compound **6** at the center and GS-441524 on the right.

Table S1. ADME Prediction of compounds **5-11** and reference compounds remdesivir and GS-441524 evaluated by on-line Server Swiss-ADME (http://www.swissadme.ch/)

Molecule	Remdesivir	GS-441524	5	6	7	8	9	10	11
**Formula**	$C_{24}H_{29}N_6O_8P$	$C_{12}H_{13}N_5O_4$	$C_{12}H_{13}ClN_4O_4$	$C_{17}H_{24}N_6O_4$	$C_{13}H_{17}N_5O_4$	$C_{14}H_{17}ClN_4O_4$	$C_{12}H_{15}N_5O_4$	$C_{14}H_{19}N_5O_4$	$C_{12}H_{16}N_6O_3$
**MW**	560.50	291.26	312.71	376.41	307.31	340.76	293.28	321.33	292.29
**#Heavy atoms**	39	21	21	27	22	23	21	23	21
**#Aromatic heavy**	15	9	9	9	9	9	9	9	9
**Fraction Csp3**	0.42	0.42	0.50	0.65	0.54	0.57	0.50	0.57	0.50
**#Rotatable bonds**	12	2	4	5	5	5	4	5	4
**#H-bond acceptors**	12	7	7	8	7	7	7	7	6
**#H-bond donors**	4	4	1	1	2	1	2	2	3
**MR**	136.01	68.48	71.74	102.99	76.03	81.35	71.13	80.75	72.85
**TPSA**	213.36	149.92	99.36	105.84	111.39	99.36	125.38	125.38	128.18
**iLOGP**	3.20	0.55	2.09	2.79	2.02	2.53	1.57	2.00	1.46
**XLOGP3**	0.76	-1.41	0.26	-0.43	-0.38	1.06	-1.05	-0.25	-1.63
**WLOGP**	1.19	-1.96	0.13	-1.53	-0.67	0.91	-0.93	-0.16	-1.36
**MLOGP**	-0.43	-1.83	-0.78	-0.91	-1.15	-0.26	-1.42	-0.88	-1.83
**Silicos-IT Log P**	-1.10	-1.93	0.65	-0.49	-0.28	1.21	-0.71	-0.15	-1.03
**Consensus Log P**	0.72	-1.32	0.47	-0.11	-0.09	1.09	-0.51	0.11	-0.88
**ESOL Log S**	-3.29	-0.94	-2.00	-1.82	-1.48	-2.58	-1.05	-1.63	-0.68
**ESOL Solubility**	2.90e-01	3.32e+01	3.16e+00	5.70e+00	1.02e+01	8.96e-01	2.61e+01	7.46e+00	6.13e+01
**ESOL Solubility**	5.17e-04	1.14e-01	1.01e-02	1.52e-02	3.32e-02	2.63e-03	8.91e-02	2.32e-02	2.10e-01
**ESOL Class**	Soluble	Very sol.	Very sol.	Very sol.	Very sol.	Soluble	Very sol.	Very sol.	Very sol.
**Ali Log S**	-4.82	-1.24	-1.91	-1.33	-1.50	-2.74	-1.09	-1.92	-0.55
**Ali Solubility**	8.48e-03	1.69e+01	3.87e+00	1.77e+01	9.81e+00	6.23e-01	2.36e+01	3.82e+00	8.21e+01
**Ali Solubility (mol/l)**	1.51e-05	5.80e-02	1.24e-02	4.70e-02	3.19e-02	1.83e-03	8.04e-02	1.19e-02	2.81e-01
**Ali Class**	Moderately	Very sol.	Very sol.	Very sol.	Very sol.	Soluble	Very sol.	Very sol.	Very sol.
**Silicos-IT LogSw**	-3.98	-0.26	-2.45	-1.97	-2.28	-2.87	-1.49	-1.91	-1.80
**Silicos-IT Solubility**	5.86e-02	1.60e+02	1.10e+00	4.08e+00	1.61e+00	4.57e-01	9.53e+00	3.95e+00	4.63e+00
**Silicos-IT Solubility**	1.04e-04	5.48e-01	3.52e-03	1.08e-02	5.25e-03	1.34e-03	3.25e-02	1.23e-02	1.58e-02
**Silicos-IT class**	Soluble	Soluble	Soluble	Soluble	Soluble	Soluble	Soluble	Soluble	Soluble
**GI absorption**	Low	Low	High	High	High	High	High	High	Low
**BBB permeant**	No	No	No	No	No	No	No	No	No
**Pgp substrate**	Yes	No	No	Yes	No	No	No	Yes	No

Molecule	Remdesivir	GS-441524	5	6	7	8	9	10	11
**CYP1A2 inhibitor**	No	No	Yes	Yes	Yes	Yes	Yes	No	Yes
**CYP2C19 inhibitor**	No	No	No	No	No	No	No	No	No
**CYP2C9 inhibitor**	No	No	No	No	No	No	No	No	No
**CYP2D6 inhibitor**	No	No	No	No	No	No	No	No	No
**CYP3A4 inhibitor**	Yes	No	No	No	No	No	No	No	No
**log Kp (cm/s)**	-9.18	-9.08	-8.02	-8.90	-8.44	-7.63	-8.83	-8.44	-9.24
**Lipinski #violations**	2	0	0	0	0	0	0	0	0
**Ghose #violations**	2	1	0	1	1	0	1	0	1
**Veber #violations**	2	1	0	0	0	0	0	0	0
**Egan #violations**	1	1	0	0	0	0	0	0	0
**Muegge #violations**	2	0	0	0	0	0	0	0	0
**Bioavailability Score**	0.17	0.55	0.55	0.55	0.55	0.55	0.55	0.55	0.55
**Synthetic**	5.96	3.89	3.44	4.17	3.73	3.67	3.57	3.80	3.48
